# Ultrasound-driven mechanical immunomodulation enhances tumor treatment sensitivity: advances from tumor mechanical immunobiology to immunotherapy applications

**DOI:** 10.3389/fimmu.2026.1830490

**Published:** 2026-06-04

**Authors:** Zhuoqi Zeng, Xiaowen Liang, Zichao Liu, Meng Du, Zhiyi Chen

**Affiliations:** 1 Key Laboratory of Medical Imaging Precision Theranostics and Radiation Protection, College of Hunan Province, the Affiliated Changsha Central Hospital, Hengyang Medical School, University of South China, College of Hunan Province, Changsha, Hunan, China; 2 Institute of Medical Imaging, Hengyang Medical School, University of South China, Hengyang, Hunan, China; 3 Institute for Future Sciences, University of South China, Changsha, Hunan, China; 4 Department of Ultrasound Medicine, Department of Medical Imaging, The Affiliated Changsha Central Hospital, Hengyang Medical School, University of South China, Changsha, Hunan, China; 5 School of Computer Science and Engineering, Central South University, Changsha, Hunan, China; 6 The Seventh Affiliated Hospital, Hunan Veterans Administration Hospital, Hengyang Medical School, University of South China, Changsha, Hunan, China

**Keywords:** cavitation, mechanical bioeffects, mechanical cues, tumor immunotherapy, tumor microenvironment, ultrasound

## Abstract

Tumor immunotherapy is often limited by microenvironmental heterogeneity and immune tolerance. The mechanical properties of tumors, such as matrix stiffening and elevated interstitial fluid pressure, sustain immunosuppressive programs and create physical barriers that restrict the infiltration of drugs and immune cells, leading to poor therapeutic responses. Targeting these mechanical constraints has thus emerged as a key strategy for sensitizing tumors to immunotherapy. As a modality that generates mechanical stimuli, ultrasound can modulate immune cells and the tumor microenvironment, offering a noninvasive and clinically promising approach to deliver programmable mechanical stimuli. By generating mechanical stimuli through acoustic radiation forces, acoustic streaming, and cavitation, ultrasound provides a basis for linking acoustic parameters with immunophenotypic outcomes. Current evidence supports two principal mechanisms through which ultrasound modulates immune responses: first, by directly regulating immune cell behavior via mechanosensitive channels—enhancing calcium signaling, promoting integrin-mediated adhesion, and triggering cytoskeletal remodeling; second, by indirectly boosting immunity through remodeling the tumor microenvironment-improving vascular permeability, loosening physical barriers, and alleviating hypoxic and metabolic stress. In this review, we summarize recent advances in ultrasound-driven mechanical immunomodulation in tumor treatment, with a focus on its bioeffects on immune cells and the tumor microenvironment, as well as the underlying molecular mechanisms revealed by advanced multi-omics techniques. Future efforts to address challenges such as dosimetry standardization, cavitation reproducibility, targeting accuracy, balancing efficacy with safety, and cell-type-specific heterogeneity will help optimize the synergy between ultrasound and immunotherapy and accelerate its clinical translation.

## Graphical Abstract

**Figure:**
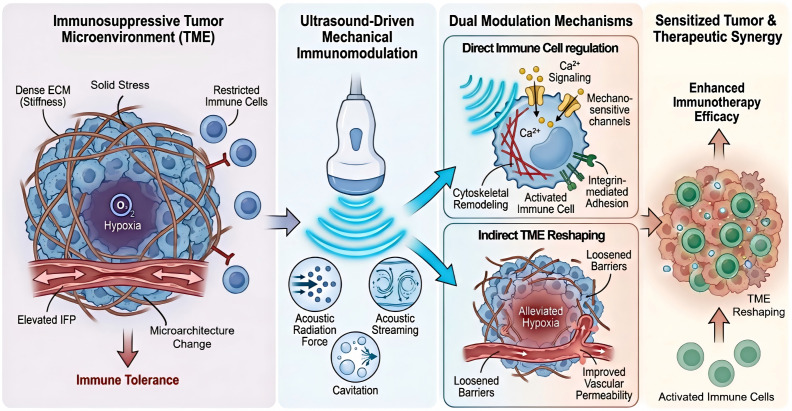

## 1 Introduction

Recent years have witnessed remarkable advances that represent landmarks in tumor immunotherapy (1), but its clinical efficacy is often limited by challenges such as microenvironmental heterogeneity and immune tolerance. Tumors often evade immune surveillance through impaired antigen presentation, upregulation of immune checkpoint pathways, and metabolic reprogramming within the tumor microenvironment (TME) (2–4). Crucially, these biochemical barriers are reinforced by a distinct layer of physical barriers. A growing body of work shows that immune responses are shaped not only by molecular cues but also by mechanical forces and tissue stress. These forces regulate cell morphology, signaling, and fate via mechanotransduction, thereby influencing immune cell trafficking and effector function (5, 6). The emerging field of mechano-immunology highlights the need to integrate physical and biochemical perspectives when designing next-generation cancer immunotherapies (7). In solid tumors, matrix stiffening, elevated interstitial fluid pressure (IFP), solid stress, and aberrant interstitial flow can sustain immunosuppressive programs while creating physical barriers that restrict drug transport and immune cell infiltration, ultimately weakening therapeutic responses (8). Consequently, targeting these mechanical constraints has gained attention as a potential strategy to enhance the effectiveness of immunotherapy.

Therapeutic ultrasound has evolved from a diagnostic adjunct into a versatile interventional tool in oncology, extending beyond thermal ablation toward the use of mechanical bioeffects, including acoustic radiation force, cavitation, and acoustic streaming (9, 10). These capabilities are closely aligned with the emerging concept of mechano-immunology, which describes how mechanical cues shape immune behavior in the tumor microenvironment (11). In this review, mechanical immunomodulation is used as an operational term for the deliberate regulation of immunity by mechanical stimuli (12), whereas ultrasound is discussed as one specific means of achieving such immunomodulation through programmable acoustic radiation force (10). This concept is distinguished from the broader term sono-immunotherapy, which encompasses ultrasound-assisted immune effects mediated by both thermal and nonthermal mechanisms (13). Within this framework, acoustic exposure can generate local mechanical perturbations, which ultimately produce mechanical bioeffects, defined here as nonthermal biological responses induced by mechanical perturbation (10). Because ultrasound can deliver tunable, noninvasive mechanical stimuli within tumors, it provides a unique means of overcoming physical barriers to immune-cell infiltration and drug delivery, thereby enhancing synergy with immunotherapy, improving intratumoral transport, and sensitizing tumors to treatment (14, 15). Therefore, it is crucial to evolve ultrasound from a general therapeutic tool into a controlled, precise immunomodulator.

This review focuses on a central question: how ultrasound-mediated mechanical bioeffects overcome physical barriers in tumors and thereby reshape antitumor immunity. First, the review delineates how tumor mechanical abnormalities create physical barriers that restrict immune infiltration, and argues that relieving these constraints requires a controllable means of delivering mechanical stimuli *in vivo*. Ultrasound offers a promising solution, yet current evidence linking its physical effects to immune outcomes remains fragmented across modalities and dosing strategies. Next, we summarize how different ultrasound parameter regimes and technological modalities can reshape the tumor immune microenvironment by disrupting or reorganizing physical barriers, modulating vascular permeability, and relieving interstitial fluid pressure, thereby enhancing immune infiltration and reshaping the immune niche. We also discuss how multi-omics analyses have begun to reveal that ultrasound physical signals can reprogram the immune microenvironment through immune-related pathways, offering new insights into the mechanosensitive immune regulatory network. Finally, this article systematically reviews the key challenges in clinical translation of this field and discusses future research directions.

## 2 Tumor mechanical immunobiology

**Figure 1:**
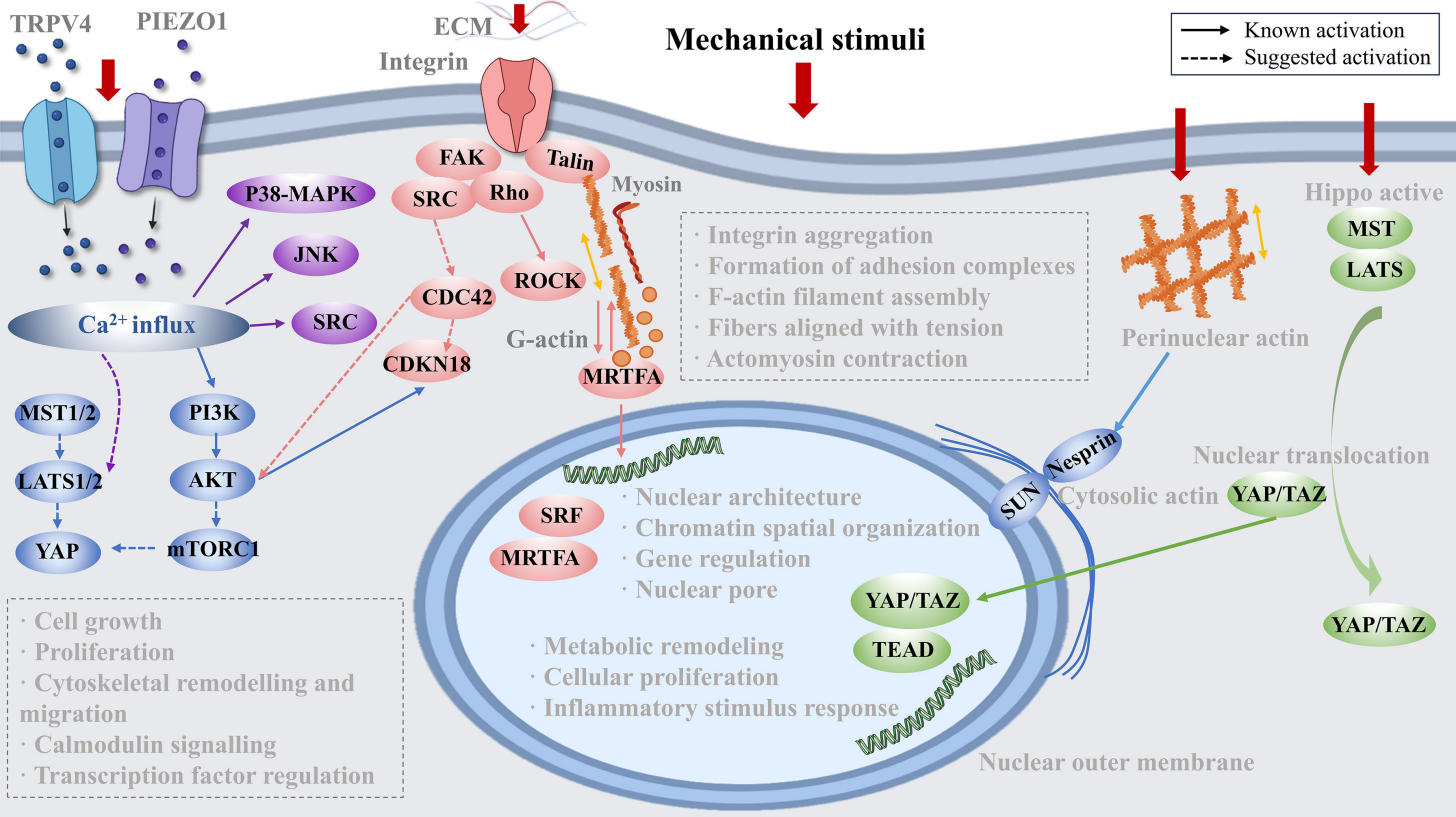
Schematic of mechanotransduction mechanisms in tumor cells (created with biorender.com).

To understand how ultrasound can overcome physical barriers, we first examine how these barriers arise and affect immune cells. The progression and therapy resistance of solid tumors are governed not only by biochemical signals but also by the abnormal biomechanical properties of the TME. Cells sense diverse mechanical cues, including tension, compression, extracellular matrix (ECM) stiffness, hydrostatic pressure, and shear stress, through surface mechanoreceptors (16). These receptors, together with focal adhesion kinase, talin, and kindlin, link the extracellular environment to the cytoskeleton and initiate mechanotransduction, thereby shaping cellular behavior and tissue morphogenesis, persistent mechanical stress or failed adaptation may disrupt tissue homeostasis and genomic integrity (**Figure 1**) (17).

Mechanical stimuli applied to the plasma membrane or transmitted from the extracellular matrix are sensed by the mechanosensitive ion channels TRPV4 and PIEZO1, as well as by integrin-based adhesion complexes. Channel activation induces Ca²^+^ influx, which modulates multiple downstream signaling pathways, including PI3K-AKT-mTORC1, SRC, JNK, and p38-MAPK, thereby influencing cell growth, proliferation, cytoskeletal remodeling, migration, calmodulin signaling, and transcription factor regulation. In parallel, integrin engagement promotes integrin clustering and adhesion-complex formation, which activate the FAK-SRC-Rho-ROCK-myosin axis and promote F-actin assembly, fiber alignment, and actomyosin contraction; under some conditions, integrin-associated signaling may also engage CDC42, which can upregulate CDKN1B to influence cell-cycle progression. Changes in actin dynamics release MRTF-A from G-actin, allowing its nuclear translocation and cooperation with SRF in regulating nuclear architecture, chromatin organization, gene expression, and nuclear pore regulation. In addition, mechanical regulation of the Hippo pathway controls YAP/TAZ localization: activation of MST1/2 and LATS1/2 promotes YAP/TAZ phosphorylation and cytoplasmic retention, whereas Hippo inhibition under mechanical stress favors nuclear translocation of YAP/TAZ, where they cooperate with TEAD to regulate transcriptional programs involved in metabolic remodeling, proliferation, and inflammatory responses. YAP signaling is increased in compressed or confined cancer cells, although the upstream mechanisms responsible for this response under solid stress remain incompletely defined. In this figure, solid lines indicate established interactions, whereas dashed lines indicate suggested pathway connections that have not yet been fully confirmed in mechanically stressed tumor cells.

### 2.1 Abnormal mechanical properties of tumors

**Figure 2:**
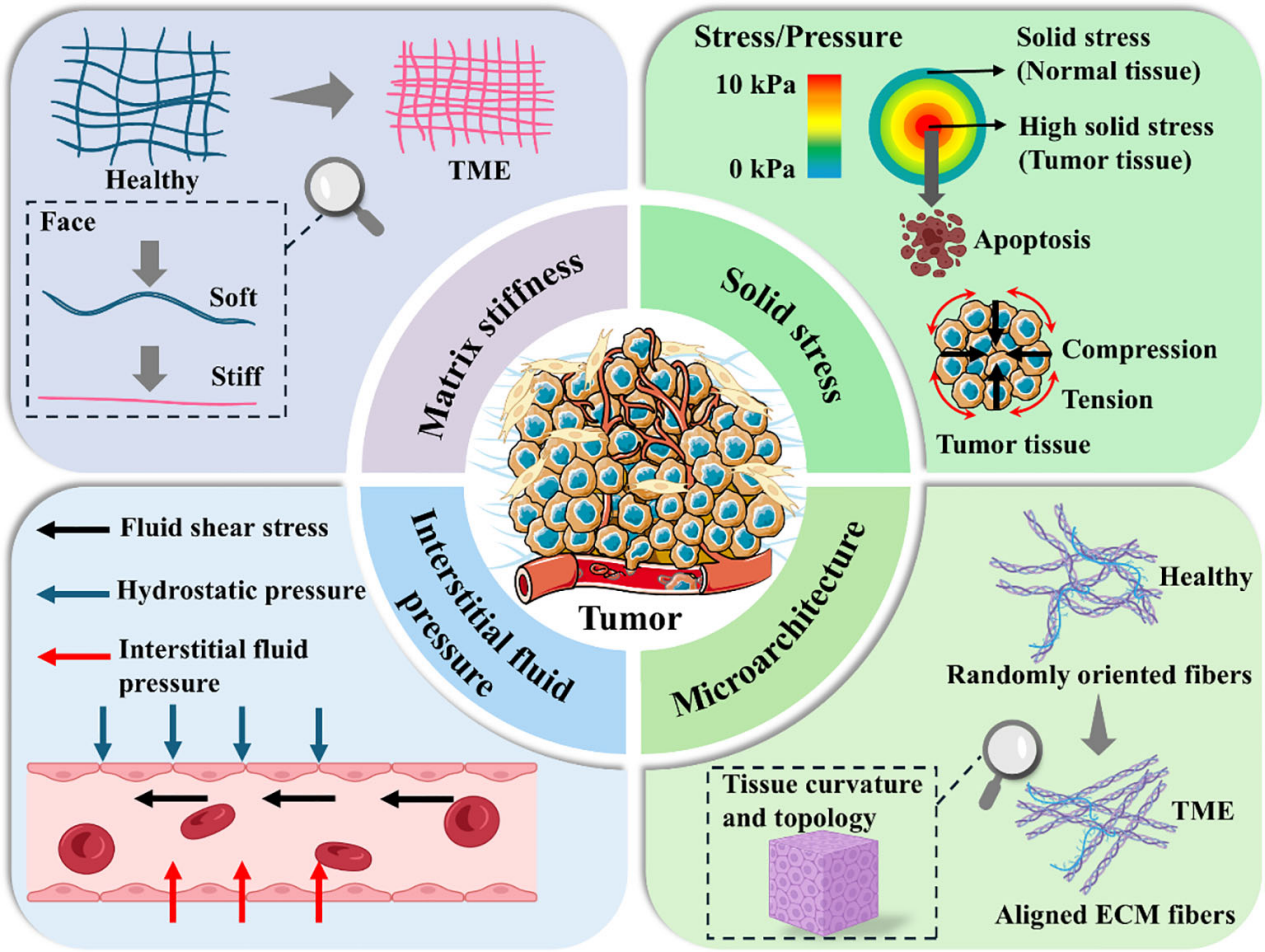
Aberrant mechanical cues in the TME (created with biorender.com).

Solid tumors develop a markedly abnormal and spatially heterogeneous mechanical microenvironment during their growth, with typical phenotypic features including: ECM stiffness, solid stress, IFP, and microarchitecture (**Figure 2**) (8). During tumor growth, cancer cells are constantly subjected to a variety of physical stresses in the surrounding microenvironment, including compression of surrounding tissues as the tumor tissue expands, changes in fluid flow within the tumor, changes in the “elasticity” of the surrounding tissues, and changes in physical stresses during tissue remodeling (18). These physical signals are captured by mechanoreceptors on cancer cells, transduced into chemical signals via structures such as the cytoskeleton, and transmitted to the cell nucleus. This process alters gene expression programs and drives invasion, metastasis, immune evasion, and therapy resistance. Together, they form a reinforcing feedback loop that accelerates tumor progression (19, 20).

Tumor tissues display four major mechanical abnormalities: increased ECM stiffness due to excessive deposition and crosslinking, solid stress from proliferating tumor and stromal cells that compress vessels and surrounding tissues, elevated IFP and shear stress caused by vascular compression and impaired lymphatic drainage, and altered ECM microarchitecture, including fiber thickening, alignment, and topographical changes. Together, these mechanical cues promote invasion, metastasis, immune evasion, and therapy resistance.

#### 2.1.1 Matrix stiffness

Matrix stiffness, typically quantified by the elastic modulus of the ECM, represents a critical determinant of tumorigenesis, as it dynamically regulates cellular behavior (21). In hypoxic tumors, excessive deposition and crosslinking of extracellular matrix proteins by cancer-associated fibroblasts (CAFs) increase tissue rigidity, making CAFs key regulators of tumor mechanical properties through dynamic ECM remodeling (22, 23). Matrix stiffness plays a multifaceted role in tumor progression, not only promoting cancer cell invasion and metastasis but also modulating immune evasion (24, 25). For example, in hepatocellular carcinoma, a stiff matrix upregulates PD-L2 and suppresses ferroptosis, thereby promoting drug resistance (26). Conversely, targeting CAF metabolism or reducing ECM stiffness can restore immune infiltration and sensitize tumors to therapy (27). These findings highlight matrix stiffness as both a hallmark of tumor progression and an actionable physical barrier. Thus, targeting ECM stiffness or CAF-mediated remodeling may help breach this barrier and facilitate immune infiltration (28, 29).

#### 2.1.2 Solid stress

Solid stress arises from the expansion and proliferation of tumor and stromal cells, which compress surrounding tissues, blood vessels, and lymphatics (30, 31). This compression reduces perfusion, impedes drug delivery, and restricts immune cell infiltration, thereby contributing to immune evasion and therapy resistance (32). Solid stress can be divided into endogenous stress, generated within the tumor itself, and exogenous stress, exerted by the surrounding host tissue (31). Endogenous stress persists even after resection, whereas exogenous stress is relieved after tumor removal (33, 34). Importantly, recent advances in intraoperative measurement and finite-element modeling have enabled quantitative assessment of solid stress in tumors and support its value as a clinically relevant mechanical biomarker (35). Overall, solid stress is not only a consequence of tumor growth, but also a direct physical barrier to transport and immune access.

#### 2.1.3 IFP

Abnormally leaky tumor vasculature leads to fluid accumulation and increased IFP (36). The resulting pressure gradients drive interstitial fluid flow toward the tumor periphery, generating shear stress that influences cell behavior (37, 38). Although high shear stress may under some conditions induce cell-cycle arrest in tumor cells (39), the dominant effect of elevated IFP in tumors is detrimental. Specifically, high IFP creates a pressure barrier that impedes convective drug delivery, while associated fluid shear stress can promote tumor-cell migration, invasion, and metastasis (40–42). Reducing IFP can therefore re-establish the transvascular pressure gradient, restoring efficient transport of therapeutic agents and immune cells into the tumor core (43).

#### 2.1.4 Microarchitecture

In addition to bulk stiffness, the microstructural organization of the ECM also strongly influences cell behavior. Tumor progression is often accompanied by collagen fiber thickening, realignment, and changes in porosity (19). These topological alterations remodel force transmission: aligned collagen fibers at the tumor–stroma interface provide “contact guidance” for tumor invasion (44), whereas ECM densification reduces porosity and forms a physical cage that restricts immune-cell migration. When pore size falls below the threshold required for nuclear passage, immune cells can become mechanically trapped and fail to infiltrate tumor nests (45). Experimental hydrogels that mimic tumor ECM architecture further show that modifying fiber organization can reshape tumor-stroma interactions, improve vascular perfusion, promote immune-cell infiltration, and restore treatment sensitivity (46). These findings indicate that microarchitecture is not merely a structural feature, but an active regulator of barrier function in tumors.

### 2.2 Mechanical regulation of immune cells in the TME

Evidence remains limited regarding how tumor-infiltrating immune cells respond to the heterogeneous mechanical features of tumors. In the TME, T cells, macrophages, NK cells, and dendritic cells sense diverse mechanical cues and adjust their behavior through mechanosensing and mechanotransduction. Recent immune cell-focused studies indicate that Integrin-FAK signaling directly regulates immune-cell adhesion, migration, phagocytosis, and cytoskeletal remodeling, particularly in macrophages and T cells. By contrast, YAP/TAZ functions as a mechanosensitive transcriptional module in immune cells, linking matrix stiffness to macrophage polarization, T-cell activation/exhaustion, NK-cell dysfunction, and dendritic-cell impairment. However, these cues can also contribute to immunosuppression by restricting immune cell infiltration and promoting dysfunctional or suppressive immune phenotypes (47). Clarifying these interactions is important for connecting tumor biomechanics to immunomodulation and for informing strategies that improve immunotherapy efficacy.

#### 2.2.1 T cells

T cells are highly sensitive to mechanical cues. While a certain degree of stiffness is required for effective T-cell receptor (TCR) triggering, the supraphysiological stiffness found in tumors is largely deleterious. Elevated matrix stiffness drives T cell exhaustion by triggering the FAK-mediated PI3K/Akt axis, thereby suppressing both proliferative capacity and effector functions (48). Densely crosslinked collagen networks further regulate T-cell fate by promoting regulatory T-cell (Tregs) differentiation via LAIR1 signaling (49)_ENREF_21 and by physically excluding T cells through steric hindrance when the matrix pore size is less than 2 μm² (45). These findings suggest that targeting stiffness and the ECM architecture could improve T-cell-based immunotherapies. Interestingly, some studies have also reported that increased tumor-cell stiffness may transiently increase the T-cell killing capacity, yet a subset of tumor cells can survive and repopulate the tissue. This paradox highlights the need for further investigation into the complex role of ECM regulation in tumor stiffness and immune responses (50). Beyond stiffness, solid stress generated by tumor growth compresses blood vessels, leading to impaired perfusion and a hypoxic microenvironment (51). Studies have shown that solid stress physically compresses high endothelial venules (HEVs), reducing their density, disrupting vascular structure, and downregulating adhesion molecules and chemokines. This directly impedes CD8^+^ T cell extravasation and establishes immune exclusion (52). In parallel, elevated IFP not only supports tumor progression but also substantially compromises the cytotoxicity and antitumor efficacy of CAR-T cells (53). Furthermore, fluid shear stress acts as a mechanical checkpoint, selectively permitting only high-avidity T cells to sustain stable contact with tumor cells (54). Ultimately, ECM stiffening and microstructural remodeling aggravate this immunosuppressive milieu; specific matrix components such as high-density collagen IV can directly induce T cell exhaustion, contributing to the formation of a “cold” immune phenotype (55).

#### 2.2.2 Macrophages

Macrophages exhibit remarkable plasticity in response to mechanical cues, which can shape their roles in inflammation, tissue repair, and tumor progression (56). Mechanical cues within the TME drive macrophage polarization via YAP/TAZ, Rho/ROCK, FAK, and Wnt/β-catenin pathways, thereby shaping tumor progression and immunotherapy outcomes (57, 58). The mechanical stress within the TME is closely linked to macrophage polarization and represents a critical driver of tumor progression and immune evasion. Accumulating evidence indicates that macrophages tend to differentiate toward the tumor-promoting M2 phenotype under conditions of high extracellular matrix stiffness, whereas a softer matrix environment is more conducive to steering their polarization toward the antitumor M1 phenotype (59). Macrophages skewed toward the M2 phenotype contribute to the formation of an immune barrier by secreting immunosuppressive factors such as IL-10 and TGF-β, which promote angiogenesis, enhance tumor cell migration and invasion, and restrict the infiltration and effector functions of CD8^+^ T cells (60). Finally, macrophage matrix clearance depends on cytoskeletal architecture, with stress fibers and podosome-associated structures coordinating extracellular matrix endocytosis and lysosomal processing, and vimentin supporting cytoskeletal balance and integrin-mediated matrix degradation (61). A low fluid shear stress environment facilitates the accumulation of TAMs and the secretion of factors such as VEGF, thereby accelerating tumor vascularization and malignant progression (62). However, mechanical regulation is multidimensional; beyond bulk stiffness, ECM topology and microarchitecture also critically influence macrophage fate. Notably, distinct from the effects of stiffness alone, certain porous and compliant 3D environments can drive M2-like polarization through spatial confinement and cell-shape modulation. This highlights the complexity of biomechanical signaling and provides a rationale for utilizing engineered materials to precisely reprogram macrophage responses (63).

#### 2.2.3 NK cells

Tumor-associated mechanical stress imposes a physical checkpoint that suppresses NK cell-mediated killing, blunting innate immune surveillance within solid malignancies. Matrix stiffness has emerged as a critical mechanical checkpoint that restrains NK cell effector functions. This biomechanical signal is transduced primarily through integrin-YAP/TAZ signaling cascades, which orchestrate extensive cytoskeletal reorganization and ultimately culminate in impaired cytotoxic capacity and diminished tissue infiltration efficiency (64). Furthermore, the dense collagen barriers associated with stiffness physically exclude NK cells from contacting tumor cells (65). Fluid shear stress can enhance NK-cell activity via NKG2D mechanosensing, aiding the elimination of circulating tumor cells (66). Current research has primarily focused on substrate stiffness and fluid shear stress, whereas the direct effects of solid stress and microstructural changes on NK cell function remain insufficiently characterized.

#### 2.2.4 DCs

As professional antigen-presenting cells, DCs integrate mechanical signals to regulate migration, maturation, and antigen presentation (67). In the TME, DCs sense physical cues via integrin adhesions, Piezo1, and nuclear tension, engaging the Arp2/3-WASP cytoskeletal machinery and the cPLA2-PGE_2_-NF-κB and Piezo1-TAZ pathways to modulate migration and immunoregulatory fate (68). Mechanical cues critically regulate DC migration and maturation. High stiffness (8–18 kPa) typically impairs DC function, reducing their motility and inducing cytoskeletal locking that hinders antigen capture and T-cell priming (69). Rigid 3D matrices can further suppress antigen uptake, skewing DCs toward a tolerogenic state (70). Conversely, fluid shear stress acts as a maturation signal; shear flow activates mechanosensitive ion channels to upregulate co-stimulatory molecules (71, 72). This suggests that while the static mechanical environment dominated by stiffness is suppressive, dynamic mechanical stimuli, such as the shear stress generated by ultrasound, could potentially reinvigorate DC function. For example, substrate stiffness induces a shift in DC polarization through activation of YAP/TAZ signaling, thereby suppressing their antitumor function (73, 74).

#### 2.2.5 Neutrophils

As the earliest effector cells recruited to the TME, neutrophils are intrinsically equipped to respond to extreme mechanical variations. Fluid shear stress acts as the primary checkpoint governing their function. Physiological flow supports neutrophil adhesion. In contrast, high fluid shear stress activates the mechanosensitive channel Piezo1, leading to intracellular calcium influx and calpain activation. This signaling cascade drives cytoskeletal reorganization and triggers the release of neutrophil extracellular traps (NETs) (75). By contrast, low shear forces, which are typical of poorly perfused tumor regions, are less effective at inducing NETosis but enhance neutrophil adhesion to vessel walls, thereby creating a dense immune barrier that physically blocks other immune cells (76). Mechanically induced NETs can trap circulating tumor cells or hinder drug delivery by serving as physical barriers. These NETs also facilitate metastatic seeding and shield tumor cells from cytotoxic assaults. Consequently, they represent a critical safety hazard in ultrasound-mediated tumor therapy (75). Additionally, neutrophils utilize amoeboid migration to navigate dense matrices, yet excessive solid stress (physical compression) or extreme confinement (< 2 μm pore size) can mechanically disrupt chromatin organization (77, 78). Furthermore, neutrophils are highly sensitive to contact guidance, utilizing the WASP-actin axis to follow aligned collagen fibers (79). Ultimately, these mechanically induced NETs form a fiber network that, while potentially trapping tumor cells, predominantly acts to shield them from cytotoxicity and hinder drug distribution (80, 81).

Overall, these abnormal mechanical features are not merely secondary consequences of tumor growth, but key contributors to immune exclusion, transport impairment, and therapeutic resistance. However, most studies have focused on static mechanical cues, such as matrix stiffness, surface roughness, and static strain, whereas the effects of dynamic mechanical stimulation on macrophages and innate immunity remain poorly understood (82). This difference is mechanistically important. Static pathological stress usually causes sustained membrane tension and prolonged activation of mechanosensitive channels. By contrast, ultrasound-mediated dynamic stress more often causes transient and reversible membrane oscillations with periodic channel gating (83). Mechanosensitive channels such as PIEZO1 may therefore respond differently to sustained and oscillatory stimuli, leading to different downstream effects. Static and dynamic inputs may also be integrated over different timescales. Static stress may favor chronic transcriptional reprogramming, whereas dynamic ultrasound stimulation may induce transient calcium pulses and short-lived signaling responses. In addition, prior pathological stress may create mechanical memory and thereby alter subsequent responses to therapeutic stimulation (84). How immune cells distinguish between these two forms of mechanical input remains a key mechanistic gap. To fill this gap, an intervention capable of delivering controlled dynamic stimuli directly to the tumor is highly desirable. Ultrasound offers a noninvasive, programmable means to achieve this via tunable mechanical stimuli. Nevertheless, how ultrasound-driven mechanical stimuli translate into specific immune outcomes still requires clarification. The following sections will examine how ultrasound-driven mechanical bioeffects overcome physical barriers and reshape antitumor immunity.

## 3 Nonthermal ultrasound mechanical bioeffects

Against this background, ultrasound is particularly well suited for overcoming tumor-associated mechanical barriers. Compared with pharmacological approaches that mainly act on downstream biochemical pathways, ultrasound directly targets the physical barriers. Furthermore, unlike many local physical interventions that are limited by poor penetration or lack of spatial controllability, ultrasound is noninvasive, focusable, and deeply penetrating (85). More importantly, ultrasound-driven mechanical bioeffects can regulate the TME through physical stimulation, helping to relieve physical barriers and shift the local milieu from immunosuppression toward immune activation. Mechanistically, ultrasound propagates as a high-frequency pressure wave composed of alternating compression and rarefaction cycles, thereby generating transient stress, strain, and displacement fields within tissues (86–88). As a pressure wave, it generates nonthermal mechanical perturbations in tissues, giving rise primarily to acoustic radiation force, acoustic streaming, and cavitation within the TME (89).

### 3.1 Acoustic radiation force

Acoustic radiation force arises from momentum transfer between ultrasound waves and the medium, producing a time-averaged directional force on tissues, cells, or particles within the acoustic field (90). Unlike oscillatory stress, ARF imposes a relatively sustained mechanical load. Under low-intensity pulsed ultrasound (LIPUS), this force can perturb the plasma membrane and cytoskeleton without substantial heating, thereby activating mechanotransduction pathways such as integrin-associated signaling (91). In immune-relevant contexts, ARF has been reported to influence myeloid-cell polarization through pathways including STAT1/STAT6/PPARγ (92). At the vascular level, it may also enhance microbubble-vessel interactions and alter local transport dynamics (93).

In tumor immunotherapy settings, acoustic radiation force can also alter vascular hemodynamics and microbubble distribution. Appropriate acoustic pressure may enhance tumor perfusion, improving drug penetration and immune cell infiltration (94). Excessive pressure, however, can damage microvasculature and reduce perfusion, drug delivery, and downstream immune responses (95). These findings highlight the importance of defining a safe and effective operating window for ARF-mediated immunomodulation.

### 3.2 Acoustic streaming and shear stress

Attenuation of acoustic waves can drive bulk fluid motion, termed acoustic streaming (96). This phenomenon generates hydrodynamic shear stress on cell surfaces, mimicking physiological flow. Since endothelial and circulating immune cells are intrinsically sensitive to shear. Shear stress has been linked to the activation of mechanoreceptors such as integrins (97), providing a mechanistic rationale for endothelial mechanotransduction that regulates immune cell trafficking. Increasing microbubble flow velocity has been reported to enhance the penetration of nanoparticles in solid tumors, overcoming the transport limitations imposed by elevated IFP (94). In therapeutic contexts, this bulk flow enhances the convective transport of therapeutic agents through the interstitium.

### 3.3 Cavitation

Cavitation, defined as the oscillation and collapse of gas nuclei under ultrasound exposure, represents the most potent mechanical mechanism (86). Depending on the acoustic pressure threshold, cavitation manifests in two distinct regimes with divergent biological sequelae. At lower acoustic pressures, stable cavitation predominates, in which microbubbles oscillate symmetrically around an equilibrium radius. This oscillatory behavior generates localized fluid shear fields, or microstreaming (98). This activity can increase tumor vascular permeability and enhance cellular membrane permeabilization, improving penetration of therapeutic agents across tumor barriers (99). With ultrasound-responsive cavitation nuclei, the dominant mechanical contribution shifts from bulk tissue displacement to cavitation-driven microstreaming (100). These effects underlie sonoporation, which facilitates the delivery of macromolecules while generally preserving cell viability. At higher acoustic pressures, cavitation shifts toward inertial cavitation, in which bubbles undergo violent expansion and collapse, generating shock waves and high-velocity microjets (101). This regime induces mechanical fractionation of the ECM and immediate cell lysis (102). In oncology, appropriately controlled inertial cavitation levels have been associated with increased immune infiltration and improved survival outcomes (103), highlighting the necessity of dose control when operating in strong mechanical regimes to minimize off-target damage.

Overall, cavitation is an important mechanism linking ultrasound exposure to immunomodulation. Its biological effects are highly dependent on the acoustic regime, as ultrasound frequency and cavitation-nucleus dose determine microbubble behavior in the vasculature and thereby influence downstream inflammatory signaling (104). Microbubble-mediated focused ultrasound has also been reported to alter immune-cell membrane permeability and modulate T-cell cytokine secretion profiles (105). However, cavitation *in vivo*, particularly in deep tissues, is inherently stochastic and difficult to predict. In deep-seated tumors, it does not always occur in a stable and fully controllable manner according to preset acoustic parameters, but is often influenced by tissue heterogeneity and local microenvironmental conditions. This variability may affect the reproducibility of downstream biological responses (106). Under relatively mild exposure conditions, such as low-intensity pulsed ultrasound, ultrasound is more likely to produce reversible mechanical responses, including radiation-force-related tissue displacement, membrane perturbation, and shear-stress-related signaling. These effects are therefore more closely associated with direct immune-cell mechanotransduction and relief of physical barriers (91). When ultrasound is combined with microbubbles or other cavitation nuclei, stable cavitation and microstreaming become more likely, which can transiently increase membrane permeability and support sonoporation-based molecular delivery while preserving cell viability (105). By contrast, under higher acoustic pressures or sufficient cavitation activity, inertial cavitation may become dominant. Rapid bubble expansion and collapse can generate shock waves and microjets, leading to mechanical tissue fractionation, direct tumor disruption, and indirect activation of antitumor immunity through the release of damage-associated molecular patterns (DAMPs) (107). Future studies should therefore combine acoustic parameter reporting with real-time cavitation monitoring and dosimetric assessment to better relate physical input to biological outcome.

## 4 Mechanistic basis of ultrasound-driven mechanical immunomodulation and advances in cancer therapy

**Figure 3:**
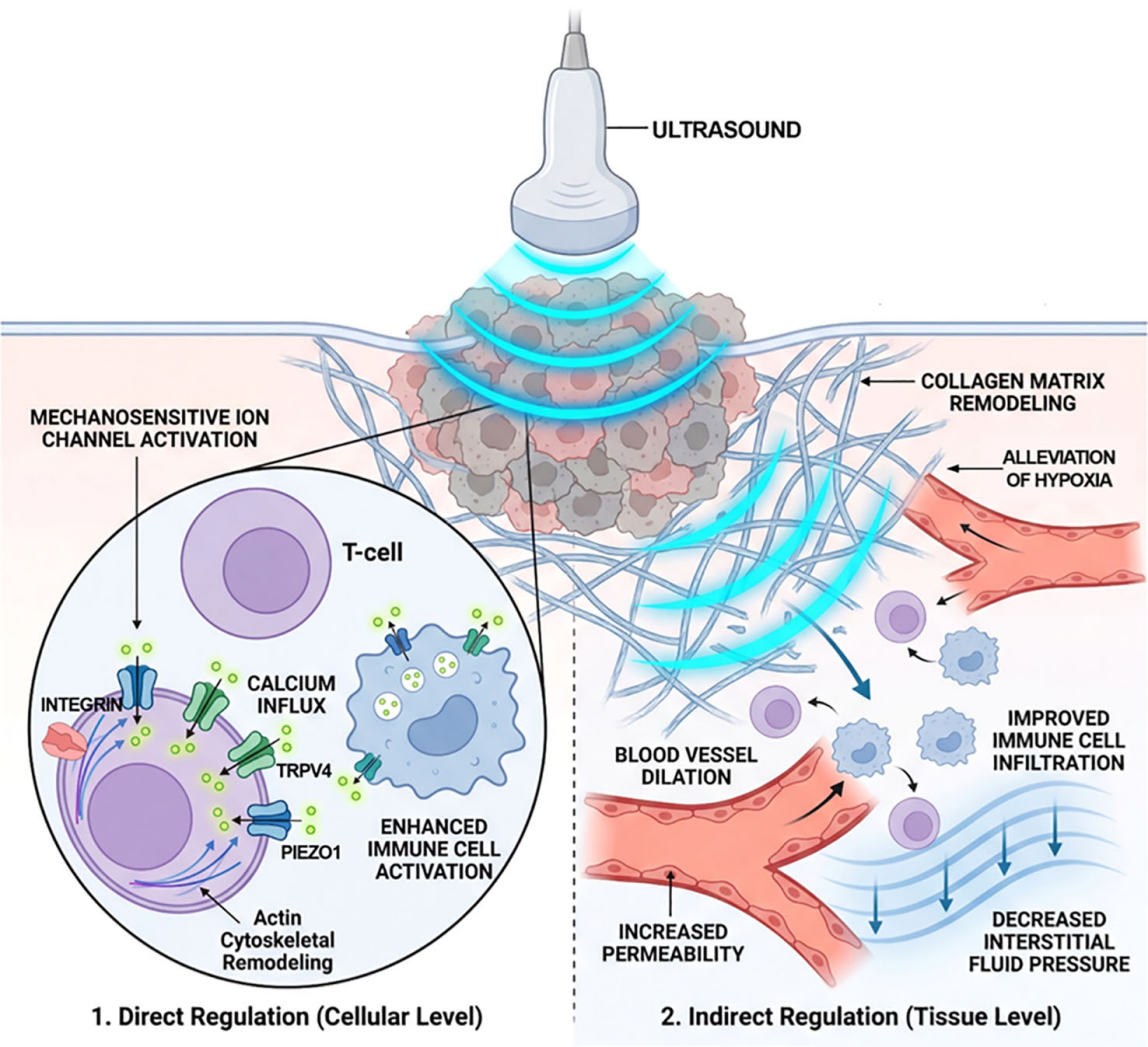
Schematic illustration of ultrasound-driven mechanical immunomodulation for sensitizing tumors to immunotherapy.

Building on the physical framework outlined above, this section examines how ultrasound-driven mechanical bioeffects are translated into immunological outcomes. In practice, the biological effects of ultrasound depend not on a single acoustic parameter, but on the dominant mechanical response generated under specific exposure conditions. Key factors include frequency, acoustic pressure or intensity, pulse duration, duty cycle, exposure time, focusing geometry, and the presence of cavitation nuclei such as microbubbles, nanobubbles, gas vesicles, or phase-change nanodroplets (108). Frequency affects tissue penetration and focusing precision, while acoustic pressure or intensity influences the strength of acoustic exposure. Pulse duration and duty cycle help determine whether mechanical or thermal effects are more prominent. Cavitation nuclei are particularly important because they lower the cavitation threshold and can shift the dominant mechanical response (109). Depending on the dominant mechanical response induced under ultrasound parameter regimes, ultrasound may enhance antitumor immunity through two major routes: direct regulation of immune-cell behavior and indirect remodeling of the TME, as shown in **Figure 3**. We first summarize the direct effects of ultrasound on immune cells and then discuss how ultrasound reshapes physical, vascular, and metabolic barriers within the TME to improve therapeutic responsiveness. Future studies should therefore combine acoustic parameter reporting with real-time cavitation monitoring and dosimetric assessment to better relate physical input to biological outcome.

The diagram depicts schematic illustration of ultrasound-driven mechanical immunomodulation for sensitizing tumors to immunotherapy. Therapeutic ultrasound produces controllable mechanical stimuli via acoustic radiation force, streaming and cavitation. It functions in two ways. Direct immunomodulation activates cellular mechanosensitive ion channels, induces calcium influx and cytoskeletal remodeling to activate immune cells and enhance their motility. Indirect remodeling breaks tumor physical barriers, optimizes vascular permeability and relieves hypoxia, enabling deep penetration of drugs and immune cells.

### 4.1 Direct regulation of immune cells

To clarify distinct biological outcomes, it is essential to differentiate between two primary modes of ultrasound-mediated direct immunomodulation. The first is intrinsic cellular mechanotransduction. Here, acoustic exposure-driven mechanical stimuli are sensed by cellular mechanoreceptors (e.g., Piezo1 channels) and converted into intracellular signals, ultimately regulating gene expression and function (110). The second is cavitation-mediated sonoporation. This involves cavitation-associated microstreaming and shear forces that temporarily and reversibly increase membrane permeability, enabling the delivery of external agents or causing widespread perturbation of membrane-proximal signaling networks. While these mechanisms may intersect under specific parameters, the dominant physical mechanism dictates the nature of the downstream cellular response.

#### 4.1.1 T cells

Studies on T cells reveal two major physical modes of ultrasound action, cell-autonomous mechanotransduction and cavitation-enhanced membrane permeability, which lead to distinct and parameter-dependent outcomes. Under mild conditions, ultrasound mainly drives regulated mechanotransduction and preferentially alters signaling and differentiation rather than causing overt membrane disruption. For example, LIPUS (1.0 MHz, 20 m W/cm², 20% duty cycle, 2h/day for 3 days) promoted a Treg-like phenotype while suppressing a Th17-like phenotype in CD4^+^ T cells through YAP/TAZ activation, indicating a differentiation-biased response at low intensity. When low-frequency ultrasound is combined with microbubbles under conditions that preserve cell viability, a moderate sonoporation effect can be introduced to enhance the delivery of immune-related molecules as an auxiliary strategy (111). Compared with LIPUS alone, this regimen still acts mainly through mechanotransduction, but microbubble-assisted amplification enables more direct and programmable gene activation. Consistently, 2 MHz ultrasound with cell-coupled microbubbles activated Piezo1 in Jurkat and primary T cells, induced calcium influx and NFAT-dependent transcription, and significantly increased reporter-gene expression after 10 min of exposure, and activation efficiency increased with stimulation duration (110). By contrast, focused ultrasound with microbubbles shifted the response toward membrane permeabilization and broader secretory remodeling. In human T cells, FUS (1 MHz, 20% duty cycle, 208–563 kPa, 2 min) in the presence of microbubbles increased membrane permeability and broadly altered the secretome, but cytokine release did not increase in direct proportion to permeability, suggesting that stronger cavitation-related effects lead to complex immune reprogramming (105). However, in both scenarios, the quantitative relationship between mechanical stimuli delivery and the resulting immune activation remains uncharacterized, underscoring the need for establishing parameter-effect correlations.

#### 4.1.2 Macrophages

Macrophages are highly sensitive to acoustic stimulation, but polarization outcomes vary depending on cell type, initial activation state, and ultrasound parameters. Current studies have shown significant differences in immune responses within disease microenvironments based on ultrasound physical input patterns. In the context of chronic inflammation, LIPUS (45 mW/cm^2^, 25 per session) promotes the transition of macrophages from an M1 to an M2 phenotype through mild mechanical signaling, characterized by downregulation of CD80/CD86 and upregulation of CD163/CD206, a process regulated by the Wnt2b/AXIN/β-catenin signaling pathway (112). This suggests that relatively mild stimulation may favor anti-inflammatory or reparative polarization. In contrast, in cancer immunotherapy, stronger or higher-frequency ultrasound is more often associated with M1-like activation. Under ultrasound irradiation (1 MHz, 1.0 W/cm², 50% duty cycle, 5 min), the piezoelectric material BFO generates a local electric field due to mechanical stress. This electrical signal directly induces the polarization of TAMs toward the M1 phenotype, characterized by upregulation of CD86, increased secretion of IL-6, IL-18, and TNF-α, and downregulation of CCL22, through NF-κB/TLR signaling pathways (113). Further mechanical interventions, combining TLR7/8 agonist R848 with mechanoluminescent nanoparticles, not only induce M1 polarization but also provide real-time feedback on the treatment effects through mechanoluminescence (114). Additionally, high-intensity directional mechanical interventions have revealed the mechanical vulnerability of macrophages; acoustic tweezers applying tensile forces via microbubbles can selectively remove drug-loaded macrophages (115). Taken together, these studies suggest that mild LIPUS conditions tend to favor anti-inflammatory or reparative programs, whereas stronger or tumor-directed regimens, particularly when combined with piezoelectric or immunostimulatory nanomaterials, are more likely to drive M1-like activation. Beyond differences in ultrasound parameters, the heterogeneous effects of ultrasound on macrophage polarization likely also reflect differences in cell origin and microenvironmental context. Macrophages such as RAW264.7 cells, THP-1-derived macrophages, and primary macrophages do not necessarily exhibit equivalent mechanosensitivity to ultrasound stimulation (116). In addition, most existing studies still rely on 2D static culture systems, which do not adequately recapitulate the complex mechanical microenvironment *in vivo*, whereas the markedly altered behavior of macrophages in 3D systems further indicates that tissue dimensionality itself is an important determinant of ultrasound responsiveness (117). Therefore, future studies should emphasize standardized acoustic reporting and integrated acoustic-mechanical-biological models to improve the comparability and interpretability of reported findings.

#### 4.1.3 NK cells

For NK cells, several reports emphasize cell-intrinsic mechanotransduction as a key mechanism under LIPUS conditions. Direct ultrasound exposure (1.0 MHz, 0.4 W/cm², 20% duty cycle, 1 min) activates Wnt/β-catenin signaling and enhances secretion of cytotoxic effectors, resulting in improved tumor-killing capacity (118). A distinct strategy uses NK-targeted piezoelectric nanomaterials, where ultrasound serves as a wireless energy source to generate coupled mechanical and electrical stimulation. In this setting, activation of the TRP-Ca^2+^ axis and cytoskeletal remodeling have been reported to enhance cytotoxicity, cytokine production, and migration (119).

#### 4.1.4 DCs

Evidence for cell-intrinsic mechanoregulation of dendritic cells (DCs) by ultrasound remains limited. Current evidence mainly supports two mechanisms. First, microbubble-assisted sonoporation enables the cytosolic delivery of antigen-encoding mRNA and immunostimulatory cargos such as TriMix, thereby promoting DC maturation and subsequent T-cell priming (120). Second, ultrasound can activate DCs indirectly through tumor-cell damage. Mechanical disruption and/or inertial cavitation may induce immunogenic cell death in tumor cells, leading to the release of damage-associated molecular patterns (DAMPs), including HMGB1 and ATP, together with surface-exposed calreticulin. These signals promote DC maturation and cross-presentation, thereby linking innate and adaptive antitumor immunity (121).

#### 4.1.5 Neutrophils

Ultrasound can exert dose-dependent mechanical bioeffects on neutrophils, but interpretation requires careful separation of intended modulation from injury. High mechanical index exposures (MI ≈ 1.0-1.8) have been reported to induce apoptosis and rupture, whereas lower-intensity regimes can increase membrane permeability while preserving metabolic activity (122). Given their sensitivity to shear stress, neutrophils may undergo Piezo1-mediated Ca²^+^ influx and NETosis under inappropriate acoustic conditions. Such mechanically induced NETs can trap circulating tumor cells or impede drug transport, acting as physical barriers (75). Ultrasound-driven neutrophil activation and NET formation may participate in early inflammatory amplification and local immune microenvironmental remodeling under certain conditions. At the same time, this response may also carry important safety risks. In particular, excessive or sustained mechanically induced NETosis could facilitate metastatic dissemination by trapping circulating tumor cells and could reduce effective antitumor clearance by shielding tumor cells from CD8^+^ T-cell- and NK-cell-mediated cytotoxicity (123). These observations suggest that the biological significance of neutrophil-related ultrasound effects is likely context dependent, and that the extent, duration, and downstream consequences of NET formation should be carefully evaluated when assessing both efficacy and safety. Accordingly, future studies should clarify when ultrasound-driven neutrophil responses promote productive immune remodeling and when they instead shift toward prometastatic or immunosuppressive outcomes. A further complication is that ultrasound contrast agents can independently affect neutrophil function (e.g., respiratory burst), potentially confounding attribution to ultrasound alone (124). Accordingly, studies aiming to modulate neutrophil function should report acoustic parameters together with injury and NETosis metrics, enabling clearer discrimination between beneficial regulation and deleterious activation.

### 4.2 Indirect regulation: reshaping the TME

**Figure 4:**
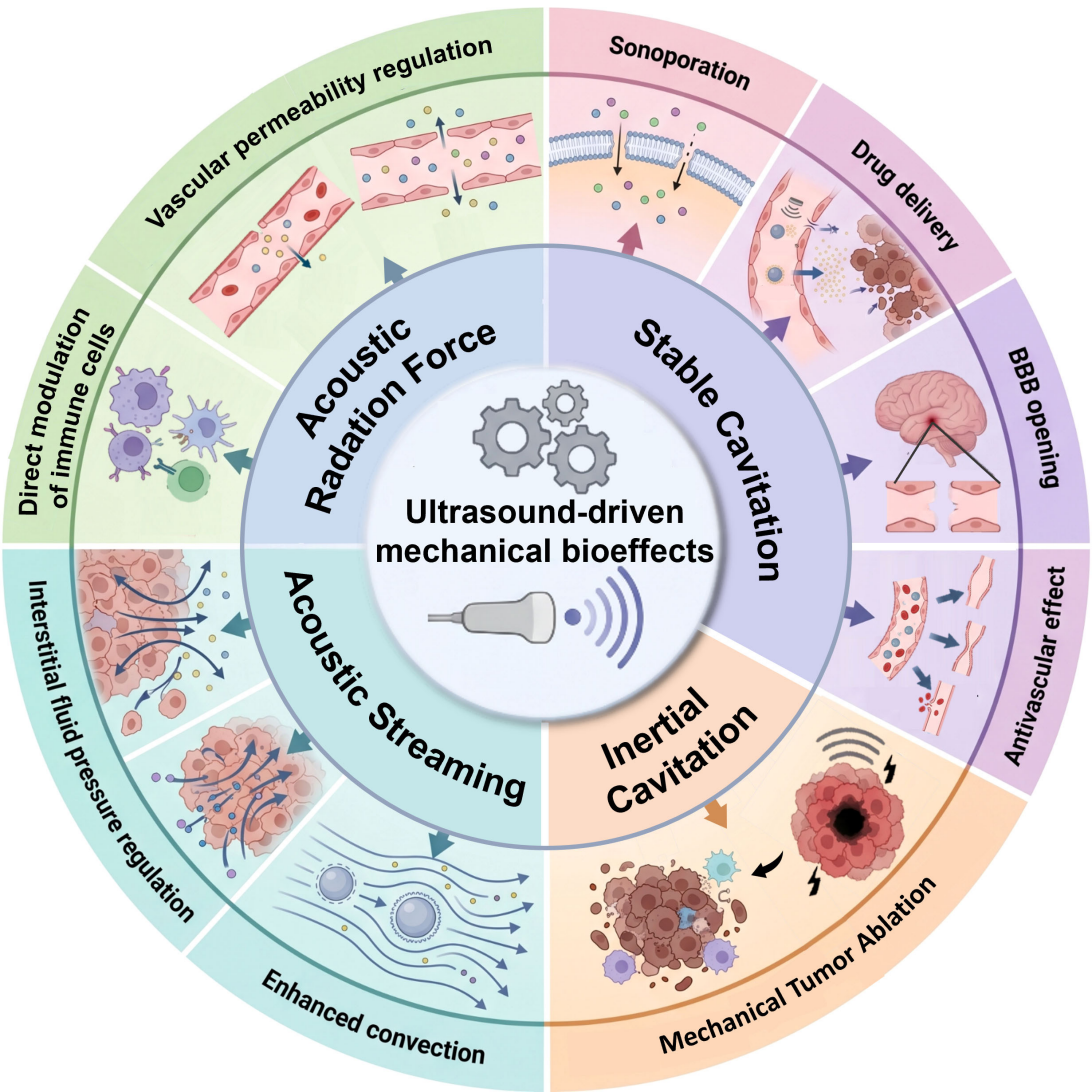
Ultrasound-driven mechanical bioeffects in tumor therapy.

The success of cancer immunotherapy relies not only on effective antitumor immune responses but is also critically constrained by multiple barriers within the TME (125). In recent years, the application of ultrasound in tumor immunotherapy has moved beyond purely thermal ablation, with mechanical bioeffects providing a nonthermal and parameter-tunable paradigm for precise TME modulation (126). By adjusting acoustic parameters, ultrasound-driven mechanical bioeffects can reshape the immunosuppressive TME through three principal pathways: loosening physical barriers, improving vascular transport and permeability, and alleviating hypoxic and metabolic stress. These effects are primarily mediated by two strategies: histotripsy, also termed mechanical high-intensity focused ultrasound (M-HIFU) or nonthermal HIFU, which drives cavitation-mediated tissue fractionation; including LIPUS-based cavitation approaches, which transiently enhance barrier permeability for drug delivery and immune activation (127). As illustrated in **Figure 4**, ultrasound-mediated mechanical bioeffects enhance tumor therapy through three interrelated mechanisms. Histotripsy induces cavitation-mediated nonthermal tissue fractionation, disrupting tumor structure and promoting antigen and damage signal release. Microbubble-assisted approaches transiently increase vascular and blood-brain barrier permeability, thereby improving therapeutic delivery and producing anti-vascular effects. In combination with radiotherapy or chemotherapy, ultrasound further enhances treatment sensitivity by increasing drug penetration and alleviating physical barriers within tumors.

The figure illustrates the hierarchical relationship between basic mechanical bioeffects and their therapeutic applications. Acoustic radiation force contributes to direct immune cell modulation and vascular permeability regulation. Stable cavitation underlies sonoporation, enabling drug delivery and transient blood–brain barrier (BBB) opening via drug-loaded, drug-coated, or targeted microbubbles; concurrently, it induces anti-vascular effects such as vascular rupture, vasospasm, and vasodilation. Inertial cavitation drives histotripsy-mediated mechanical tissue ablation, triggering tumor cell apoptosis and immune cell infiltration. Acoustic streaming enhances convective transport and helps alleviate interstitial fluid pressure.

#### 4.2.1 Loosening physical barriers

Dense ECM and elevated IFP form a physical barrier to immune infiltration (128). M-HIFU uses high-amplitude focused ultrasound pulses to generate cavitation bubble clouds, resulting in nonthermal tissue fractionation (129). In contrast to thermal HIFU (T-HIFU), which relies on heat deposition, M-HIFU uses high-amplitude pulses (>10 MPa) to induce nonthermal cavitation effects, allowing better control of bubble activity and reduced heat diffusion (107). Mechanistically, this process physically severs the tension-bearing collagen network, leading to macroscopic stress relaxation. The reduction in solid stress and IFP creates low-resistance hydraulic pathways for immune cell migration and intratumoral transport (128).

The loosening of these physical barriers may also lead to secondary immunological consequences. The resulting cellular debris serves as a source of tumor-associated antigens (TAAs) and DAMPs (e.g., ATP and HMGB1), effectively promoting antigen uptake and immune priming (130). Tissue-selective disintegration generates debris with immunostimulatory potential (131). Cavitation-cloud histotripsy (CH) and boiling histotripsy (BH) enable microscopic tissue fragmentation through distinct bubble nucleation mechanisms (132), supporting M-HIFU as a platform for precise, image-guided, and immunocompatible tumor debulking. Beyond cytoreduction, M-HIFU has been reported to enhance CD8^+^ T-cell and NK-cell infiltration, promote M1-like macrophage polarization, increase proinflammatory mediators (e.g., IFN-γ and IL-12) (133), and release tumor-derived RNA, DNA, and proteins while reducing immunosuppressive signals and enhancing DC activation (130). Mechanistically, it augments cancer immunogenicity via PANX1-mediated Ca²^+^/ATP release (134). Together, DAMP-associated signaling and enhanced antigen uptake by conventional DCs can support T-cell priming and distant tumor control (135). Additionally, M-HIFU reduces the levels of immunosuppressive factors, such as TGF-β1 and IL-10, within the tumor tissue, creating a more favorable immune microenvironment (136). Together with DAMP-associated signaling, these changes may support T-cell priming and improve local immune accessibility.

Overall, these findings suggest that the value of M-HIFU lies not only in local tumor debulking, but also in its ability to relieve mechanical confinement within tumors, thereby facilitating immune infiltration and therapeutic delivery. However, current evidence remains largely preclinical, and relief of mechanical constraints, antigen release, and downstream immune remodeling still need to be further clarified.

#### 4.2.2 Improving vascular permeability

Tumor vasculature is a major barrier to immunotherapy because of its tortuous architecture, heterogeneous perfusion, and dysregulated endothelial junctions, all of which restrict macromolecular transport and leukocyte trafficking (137). UTMD/UTND can improve intratumoral delivery and immune-cell access by using controlled cavitation to transiently increase vascular and cellular permeability (138). Mechanistically, stable cavitation and acoustic radiation force generate microstreaming and shear stress at the endothelial interface, causing reversible cytoskeletal perturbation and transient widening of inter-endothelial junctions that permit paracellular transport (139). This effect is highly dependent on acoustic dosimetry: excessive pressure may damage vascular integrity, whereas optimized protocols maximize delivery efficiency (140). Consistent with this, computational modeling by Koutsi et al. showed that combining mechanotherapy to relieve solid stress with sonopermeation achieved maximal efficacy within a narrow pressure range of 0.24-0.27 MPa (mechanical index ≈ 0.17), highlighting the need to coordinate vascular opening with relief of extravascular compression (141).

The outcome of sonopermeation is also shaped by the cavitation agent. Gas-core microbubbles remain largely intravascular and are therefore well suited for endothelial modulation and vascular opening (142, 143), whereas submicron nanobubbles may extravasate and support deeper tissue penetration (144). In addition, LIFU-activated phase-change nanodroplets can vaporize *in situ*, mechanically disrupting dense stromal structures and further facilitating transport within tumors (145).

**Figure 5:**
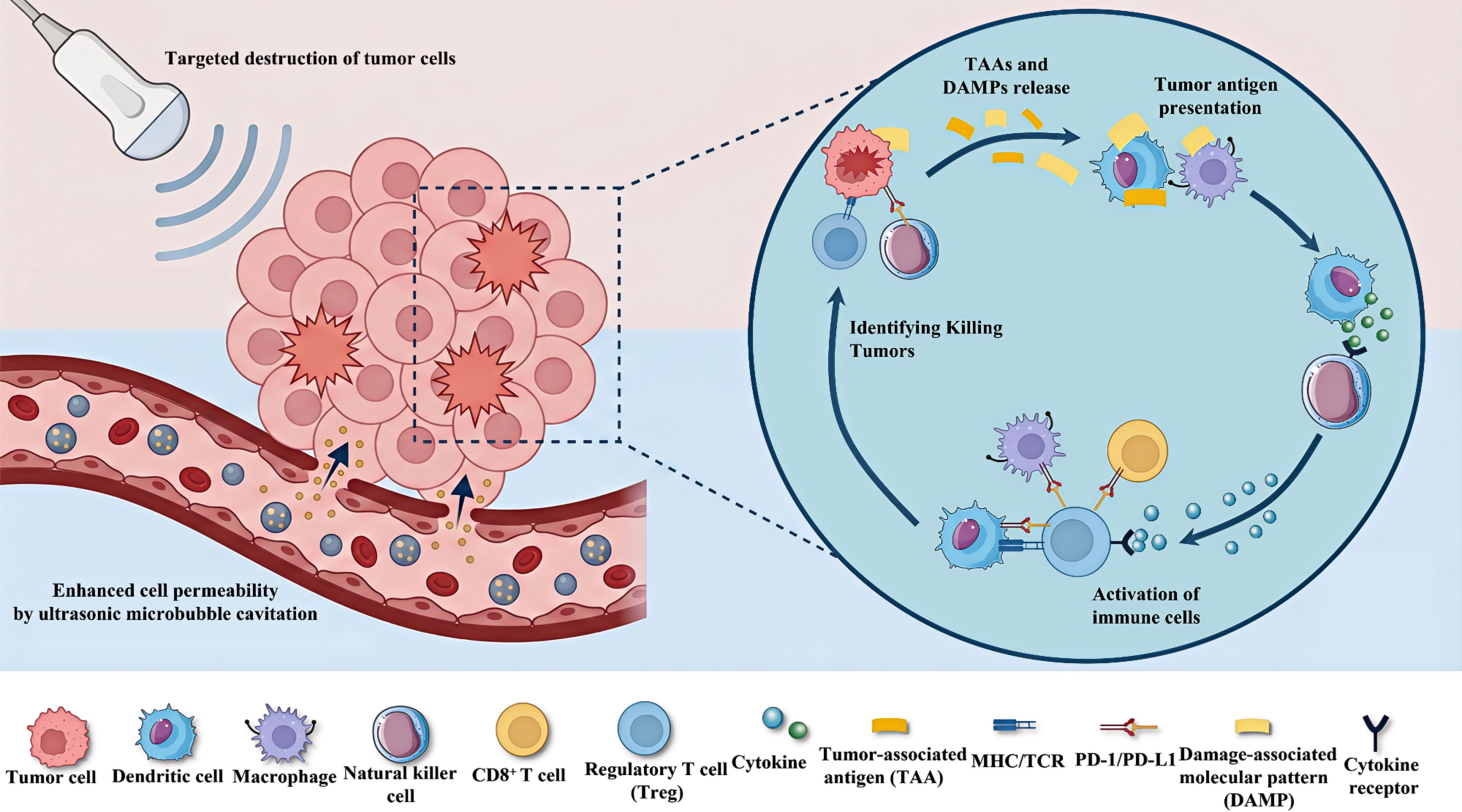
Ultrasound–microbubble synergy in tumor immunotherapy (created with biorender.com).

Beyond increasing passive permeability, sonopermeation can also enhance endothelial permissiveness to immune-cell infiltration. Mechanical stimulation of endothelial cells has been reported to increase adhesion-related signaling and chemokine expression, including CX3CL1, thereby enhancing recruitment of CX3CR1^+^ NK cells in a pressure-dependent manner (146). Rapid neutrophil recruitment and transendothelial migration have also been observed following ultrasound-mediated vascular opening (147). In parallel, cavitation-associated tumor injury or necrosis can promote the release of TAAs and DAMPs, which recruit and activate antigen-presenting and innate effector cells such as dendritic cells, macrophages, and NK cells, thereby supporting synergy with immunotherapy. **Figure 5** illustrates the synergy of ultrasound and microbubbles in tumor immunotherapy, inducing tumor cell necrosis, antigen release, and immune cell activation to boost antitumor immunity.

Low-frequency ultrasound activates microbubbles to induce cavitation and acoustic pore effects, triggering tumor cell necrosis, tissue destruction, and the release of tumor-associated antigens (TAAs) and damage-associated molecular patterns (DAMPs), and subsequently recruiting and activating DCs, macrophages, and NK cells to enhance antitumor immunity.

From a therapeutic perspective, sonopermeation offers a spatiotemporally controllable strategy for precision delivery. In tumor models, UTMD has been reported to enhance drug delivery and antitumor immune responses while maintaining acceptable safety profiles (148). Ultrasound-activated nanovesicles and nanotheranostic platforms may further strengthen this effect by reversing local immunosuppression and improving the efficacy of immune checkpoint blockade (149, 150). Blood-brain barrier opening provides a clinically advancing example of this concept. Ultrasound-mediated BBB modulation, driven by cavitation, tight-junction remodeling, and increased endothelial vesicular transport, has progressed from large-animal studies to clinical trials and has improved delivery of chemotherapeutics and therapeutic antibodies to brain tumors (151–153). This strategy has also been extended to immunomodulatory payloads such as the STING agonist cGAMP and to endothelial-targeted gene systems, including VCAM-1-directed Gasdermin-E delivery, which may restore pyroptosis sensitivity and overcome resistance to radioimmunotherapy (154, 155).

Nevertheless, tumor heterogeneity, particularly differences in vascular phenotype, matrix stiffness, and collagen density, can still limit interstitial penetration. This highlights the need to tailor carrier design and acoustic parameters to specific tumor contexts (156–158). Representative ultrasound-microbubble-enabled immunotherapy strategies are summarized in **Table 1**.

**Table 1:** Ultrasound microbubble-mediated tumor immunotherapy strategies.

Synergistic fields	Delivered substance	Microbubbles			Ultrasound exposure parameters	Tumor type	Effect	References
		Shell Core Size						
Chemotherapy drugs	DTX and R837	lipids	C_3_F_8_	/	1.0 MHz, 1.5 W/cm^2^, 3 min	Breast tumor	Remodeling the immunosuppressive microenvironment, validated in animal experiments	(175)
	PTX and L-Arg	O-CMC	PFH	300 nm	1.0 MHz, 0.5 W/cm^2^, 60 s	Hepatocellular carcinoma	Inhibits tumor growth with good biosafety	(176)
	DTX and PFP	lipids	PFH	61.63 nm	2.5 W/cm^2^, 3 min	Pancreatic ductal adenocarcinoma	Synergistic enhancement of antitumor effects	(177)
	Paclitaxel	lipids	C_3_F_8_	469.5 ± 32.87 nm	1.0 MHz, 60 s	Prostate cancer	NBs can stably carry PIX and have an antitumor effect	(178)
	Apatinib	lipids	SF_6_	/	840 kHz, 2 min, 0.75 W/cm^2^	Breast cancer	UTMD can enhance the inhibitory effect of APA on the angiogenesis of INBC	(179)
Immunosuppressants	antiPD-1 antibody	lipids	C_3_F_8_ and O_2_	1.09 ± 0.09 μm	1.0 MHz, 0.5 W/cm^2^	Colorectal cancer	Reversing the immunosuppressive microenvironment and significantly enhancing anticancer efficacy	(180)
		lipids	C_3_F_8_	1957 ± 53.37 nm	1.0 MHz, 3 W/cm^2^, 5 min	Breast tumor	LIFU-TMD inhibits tumor growth through a dual mechanism of anti-vascularity and immune activation, and significantly sensitizes ICB therapy in combination with an anti-PD-L1 antibody	(181)
Gene therapy	pDNA E-cadherin	protein	GVs	200 nm	1.0 MHz, 0.5 MPa, 1 min	Glioma	G2/M phase delivery of E-E-E-E-calmodulin efficiently inhibits tumor invasion and metastasis	(182)
	SIK2 siRNA and antimiR21	lipids PLGA	/	169.2 ± 21 nm	1.0 MHz, 40 s, 1 W/cm^2^	Ovarian cancer	SiRNA-NPs showed good transfection efficiency in cells and had good accumulation in xenograft tumors *in vivo*	(183)
	pCMV-Luciferase	Lipids albumin	C_3_F_8_	118.8 ± 3.5 229.3 ± 8.4	1.0 MHz, 5 s, 5.0 W/cm^2^	Oral squamous carcinoma	Ultrasound-mediated sonoporation induced gene transfection *in vitro and in vivo*	(184)
Tumor Vaccine	Engineered tumor cell membrane proteins, mRNAs, and Ce6	PLGA	/	200 nm	1 W/cm^2^,1 min	Breast cancer	Effective activation of antitumor immunity provides a new strategy for the prevention of tumor metastasis.	(185)
	DCs plasma membrane fragments loaded with tumor antigens	lipids	C_3_F_8_	1-6 μm	18 MHz	Breast cancer	Highly effective in targeting lymphoid organs, activating antitumor immune response, and significantly inhibiting TNBC growth	(186)

DOX, doxorubicin; R837, imiquimod; PTX, paclitaxel; PFH, perfluorohexane; O-CMC, O-carboxymethyl chitosan; L-Arg, L-arginine; PFP, perfluoropentane; PLGA, poly (lactic-co-glycolic acid); C3F8, perfluoropropane gas; O_2_, oxygen; DTX, docetaxel; E-cadherin; anti-PD-1, anti-programmed death receptor-1 antibody; pDNA/E-cadherin, plasmid DNA encoding E-cadherin; GVs, gas vesicles; Ce6, Chlorin e6; pDNA/PEI_25_, polyethylenimine (25 kDa)-plasmid DNA complex. Ultrasound parameters are presented as frequency (MHz), intensity (W/cm²), and duration (min/s);/: indicates not reported. Intensity values in this table are reported as cited in the original literature. Readers should note that different studies may use different intensity metrics (e.g., ISPPA, ISPTA) and that direct comparison of intensity values across studies should be made with caution. Standardized reporting of intensity metrics is recommended for future work.

Collectively, ultrasound-mediated vascular opening enhances not only intratumoral delivery but also immune cell trafficking and endothelial permissiveness, providing a mechanistic basis for combining UTMD/UTND with immunotherapy and targeted delivery platforms.

#### 4.2.3 Alleviating hypoxia and reversing metabolic immunosuppression

Tumor hypoxia and metabolic stress are major drivers of immunosuppression and treatment resistance. Ultrasound can counter these effects by improving perfusion, increasing oxygen availability, and reducing hypoxia-associated metabolic dysfunction. Ultrasound combined with microbubbles has been shown to enhance tumor blood flow and tissue oxygenation through nonthermal mechanisms, partly through endothelial mechanotransduction, including increased eNOS phosphorylation and subsequent elevation of nitric oxide and ATP levels (159). These effects are strongly parameter-dependent: shorter pulses with higher pulse repetition frequency tend to promote vasodilation and improve intratumoral delivery, whereas longer pulses with lower pulse repetition frequency may damage vessels and reduce perfusion (160). Accordingly, optimized acoustic regimens have been reported to increase blood flow and reduce HIF-1α expression (161).

Ultrasound can also actively promote reoxygenation. Oxygen-loaded microbubbles can release oxygen upon ultrasound stimulation, transiently increasing intratumoral oxygen tension and suppressing hypoxia-associated pathways such as HIF-1α and VEGF-A (162). Cavitation-based approaches combined with exogenous nitric oxide donors have likewise shown synergistic effects in relieving hypoperfusion (163). Beyond restoring oxygen supply, ultrasound may also reverse the metabolic consequences of hypoxia. Ultrasound-stimulated microbubble cavitation has been reported to reduce glycolysis and lactate secretion, thereby alleviating acidosis while improving perfusion (164). In parallel, mechanically induced signaling, including Piezo1-dependent Ca²^+^ influx and perturbation of metabolic regulators such as AMPK, may impose metabolic stress that selectively promotes tumor-cell death (165).

These physiological and metabolic changes can enhance antitumor immunity. In pancreatic ductal adenocarcinoma models, sonoporation with NH002 microbubbles reduced hypoxia, increased intratumoral CD8^+^ T-cell infiltration, and improved the efficacy of anti-PD-L1 therapy, resulting in prolonged survival (166). Under stronger mechanical regimens, histotripsy has also been reported to suppress hypoxia-responsive pathways and upregulate CXCL10, thereby promoting recruitment of CXCR3^+^ CD8^+^ T cells into the tumor core (167). Together, these findings indicate that ultrasound can relieve hypoxia-associated immunosuppression not only by restoring perfusion and oxygenation, but also by reshaping the metabolic conditions that limit effective antitumor immunity. This supports combining ultrasound with chemo-immunotherapy and other metabolism-oriented strategies.

Overall, ultrasound-mediated remodeling of the TME extends beyond local tumor disruption to improve immune accessibility, vascular transport, and metabolic fitness. These effects provide a rationale for combining ultrasound with immune checkpoint blockade, chemotherapy, and nanoplatform-based delivery systems, which have shown promising synergy in preclinical models. **Table 2** summarizes the immune effects of M-HIFU combination therapy across different tumors, highlighting increased CD8^+^ T cell infiltration and enhanced immune responses. The variations in tumor types and treatment parameters among the different combination strategies affect both the intensity and duration of the immune response. Notably, similar immune phenotypes can emerge from distinct mechanical pathways. Thus, ultrasound-based cancer immunotherapy requires not just stronger stimulation, but a more precise matching of acoustic regimens to the dominant mechanical process and desired immune outcome. However, significant challenges remain, such as acoustic dose standardization, cavitation reproducibility, targeting precision, and the need to balance efficacy with safety.

**Table 2:** Summary of preclinical studies on the effects of M-HIFU on antitumor immune responses.

Tumor			Combination treatment	Main findings	Year	References
Type	Size	Ablation				
Melanoma	330–400 mm^3^	40-50%	Anti-CD40 agonist antibody	>70% tumor weight reduction at 1 week; ~4-fold increase in CD8^+^ T cells; survival extended by 40 days with ICI	2020	(187)
	Over 7 mm in diameter	80-90%	Anti-CTLA-4	Tumor volume reduced 4-6× by day 18; early NK/myeloid cell peak; increased neutrophil chemotaxis and DCs in TDLN; enhanced CTL on day 10-12; ICI efficacy improved	2023	(188)
	<4000 mm^3^	20%	/	BH triggered antigen transport and DC activation within 24 h; tumor inhibition and survival benefit were observed at 15–35 days; systemic antitumor immunity was confirmed	2024	(135)
	80 mm³	31-78%	T-MBs	62.3% tumor ablation on day 1; rapid CD8^+^ T-cell infiltration; IFN-γ, TNF-α, IL-6 decreased; tumor growth slowed by day 3; 78.5% volume reduction by day 7; survival extended	2024	(189)
	657.4 mm³	83.3%	NDs-PFH& Anti-PD-L1	On day 14, tumor volume reduced by 83.3%, distant metastases by 76.6%; CD8^+^ T cells increased, Tregs decreased; TNF-α and IFN-γ elevated; αPD-1 combination amplified effect	2025	(190)
Breast cancer	2000 mm^3^	20%-40%	Anti-PD-L1	Increased CD4^+^, CD8^+^ T cells and NK cells on day 7; combination with anti-PD-L1 significantly enhanced immune response and survival on day 50	2022	(133)
	55–60 mm^3^	75%	DOX&Anti-PD-L1	Increased CTL infiltration on day 15; 88.9% tumor inhibition on day 30; 97.7% inhibition with PD-1 blockade; systemic immunosuppression alleviated	2022	(191)
	Approx. 6 mm diameter	70-80%	/	IL-6 and TNF-α elevated (4–48 h); increased tumor-infiltrating DCs and T cells (2–7 d); tumor volume reduced 3× by day 26; survival extended from 32 to 40 days	2023	(192)
	100 mm^3^	60-80%	NP-G/P	>60% tumor weight reduction on day 7; CD8^+^T cells and DCs increased 10-11×; elevated TNF-α and IFN-γ; 66.7% complete regression by day 30; survival extended to 45 days	2024	(193)
	65.45 mm³	42.5%	Anti-PD-L1&nanodroplet	By day 7, tumor growth slowed; macrophages ↑6.8×, CD8^+^ T cells ↑5.5×; median survival in combination group reached 39.5 days	2025	(194)
Pancreatic cancer	325–380 mm^3^	/	Anti-CTLA-4&Anti-PD-L1	Significant tumor growth inhibition by day 12; increased CD8^+^ T cells; survival extended by 12.5 days (17 days with ICI)	2021	(195)
	300–350 mm³	/	PFH-loaded CD133-targeted Organosilane Nanomicelles	PFH phase transition improved ablation efficiency, inducing necrosis/apoptosis within 1 week and significantly reducing tumor size in 2 weeks	2024	(196)
Neuroblastoma tumor	1200–1750 mm^3^	<2%	Anti-CTLA-4 &Anti-PD-L1	Survival rate increased to 62.5%; NK and CD8α^+^DC infiltration increased (24–72 h); enhanced T-cell responses and ICI efficacy	2020	(197)
Spontaneous tumor	/	25-50%	DOX	Partial tumor remission and increased IFN-γ^+^T cells on day 7; adriamycin cotreatment increased CD3^+^T cells and Tregs; tumor progression noted at 60 days; combination enhanced drug delivery and immune activation	2023	(198)
Cervical and ovarian cancer	80–100 mm³ and detectable solid tumors	50%-60% and 50–80 mm³	GAZn-PEG NPs	Cervical cancer: >60% tumor weight reduction, 10–11× increase in CD8^+^ T cells and DCs by day 7; 80% tumor volume reduction and survival extended to 34 days. Ovarian cancer: CD3^+/^CD8^+^ T cells and M1 macrophages increased, M2 decreased 50%, cytokines elevated; 80% volume and 55% weight reduction, survival improved by 40%	2025	(199)
Prostate cancer	250 mm^3^	/	Cholesterol-lowering treatment	CD4^+^/CD8^+^ lymphocyte activation at 24 h induced tumor-specific immunity, inhibiting tumor growth and prolonging survival (72 h-20 d); enhanced by cholesterol-lowering agents	2025	(200)

CTLs, cytotoxic T lymphocytes; Tregs, regulatory T cells; DOX, doxorubicin; NDs-PFH, perfluorohexane nanodroplets; NP-G/P, Loaded with gambogic acid and perfluorooctyl bromide poly nanoparticles; T-MBs, B7-H3-targeted microbubbles; GAZn-PEG NPs, gambogic acid (GA)-based coordination polymer-GAZn-PEG nanoparticles; TDLN, tumor-draining lymph node./, indicates not reported.

### 4.3 Multi-omics studies of ultrasound-driven immunomodulation

Research on ultrasound-driven mechanical regulation of tumor immunity remains largely descriptive, with insufficient mechanistic and quantitative linkage between physical input and immune output. Heterogeneity in ultrasound modalities, acoustic parameters, and experimental models further limits mechanistic comparison and generalizable conclusions, while conventional readouts often fail to distinguish true immune remodeling from nonspecific inflammation or delivery-related effects. Multi-omics approaches therefore offer unique value by linking physical stimuli to molecular responses and functional immune outcomes.

First, they help distinguish early inflammatory activation from functionally meaningful immune remodeling. In a study on focused ultrasound-driven blood-brain barrier opening, transcriptomic analysis identified 12 inflammation-related pathways. The researchers quantified pathway activation scores and classified sterile inflammatory responses into three grades, mild, moderate, and acute, establishing a quantitative framework to differentiate controlled inflammation from tissue damage. Correlation analysis further linked physical parameters to molecular outcomes: the extent of blood-brain barrier opening was the strongest predictor of transcriptional responses, while acoustic pressure and microbubble dose contributed differentially across pathways. For instance, complement and hypoxia pathways correlated more strongly with microbubble dose, whereas interferon-γ and inflammatory response pathways correlated more strongly with acoustic pressure (168). Thus, BBB opening intensity together with TNFα/NF-κB and apoptosis-associated programs may help distinguish controlled opening from harmful sterile inflammation. Similarly, single-cell transcriptomic profiling of pancreatic tumors after boiling histotripsy and reovirus treatment identified 30 distinct immune subsets, revealing that neutrophils expressed N2-like pro-tumor genes, CD8^+^ T cells carried exhaustion markers, and a distinct SIRPα^+^ macrophage subset was present, demonstrating that cell-intrinsic characteristics shape immune responses independently of treatment, at the single-cell level, N2-like neutrophil signatures, Treg abundance, and immune cell/Treg ratios may help distinguish beneficial antitumor immunity from suppressive immune remodeling. Combined treatments also modulated over 2,600 genes across immune clusters, far more than either treatment alone, with distinct expression patterns across cell types (169).

Second, multi-omics can identify candidate pathways for rational combination therapy. Integrative analyses combining tumor transcriptomes, drug-sensitivity datasets, and HIFU-driven expression changes have highlighted ferroptosis-related agents as potential partners and implicated the HDAC-SIRT3 axis and cytokine regulation as candidate convergence pathways (170). In addition, a simulation-based transcriptomic study suggested that ultrasound-like mechanical stress may suppress EMT and stemness-associated programs epithelial–mesenchymal transition and cancer stemness through coordinated downregulation of TGF-β, Wnt/β-catenin, Notch, and YAP/TAZ signaling, thereby revealing a mechanosensitive regulatory network that links ultrasound-like physical input to tumor cell plasticity programs (171). Beyond oncology, multi-omics analyses of ultrasound-targeted microbubble destruction in myocardial ischemia-reperfusion injury suggested that ultrasound can reshape macrophage immune-metabolic reprogramming through lactylation-related regulation, pointing to a previously underappreciated immune-regulatory network that may also be relevant to immunosuppressive metabolism in the TME (172). This finding is highly relevant to tumor immunotherapy, as lactate-related metabolic reprogramming is also a key immunosuppressive mechanism in the TME. Collectively, these advances move the field from descriptive phenotyping toward mechanism-informed therapeutic design.

Third, omics approaches may support clinical translation by enabling biomarker development and patient stratification. Emerging ultrasound radiogenomic studies are beginning to link molecular programs with noninvasive imaging phenotypes, raising the possibility of using imaging signatures, circulating biomarkers, and quantitative acoustic indicators as surrogate markers of treatment response (173). Such strategies may ultimately help tailor acoustic regimens and combination therapies to specific tumor contexts.

Taken together, the major value of multi-omics in this field is not merely to catalog ultrasound-driven changes, but to identify which molecular programs are associated with beneficial immune remodeling rather than transient sterile inflammation or nonspecific tissue stress. Future studies should therefore integrate standardized acoustic reporting, key physical dosimetry, and time-resolved, spatially resolved, and cell-type-specific omics. This will be essential for defining actionable mechanistic pathways, improving cross-study comparability, and enabling more precise clinical translation of ultrasound-enabled cancer immunotherapy.

## 5 Conclusion and prospects

Ultrasound-mediated mechanical bioeffects have emerged as a promising means of sensitizing tumors to immunotherapy by reshaping the mechanical landscape of the tumor microenvironment. Unlike passive drug carriers, ultrasound provides controllable physical inputs, ranging from acoustic radiation force to inertial cavitation, that can reduce physical barriers such as matrix stiffening and elevated interstitial fluid pressure. These effects operate through two complementary routes. Ultrasound can directly influence immune-cell behavior through mechanotransduction pathways, including channels such as Piezo1. At the same time, it can remodel vascular and stromal structures, thereby improving the penetration of checkpoint inhibitors and effector immune cells. In combination with microbubbles and drug-delivery platforms, ultrasound offers a noninvasive and scalable approach that links physical delivery enhancement with immune activation.

Clinical translation of ultrasound-driven mechanical immunomodulation still faces several major challenges. First, treatment parameters have not yet been standardized across different ultrasound systems and clinical settings, which makes it difficult to compare studies and to evaluate reproducibility and safety. Establishing unified reporting standards and reliable methods for quantifying *in situ* exposure will therefore be essential. Second, precise control of the acoustic field in deep-seated tumors remains challenging because tissue heterogeneity, acoustic attenuation, blood perfusion, and unpredictable cavitation behavior can all affect treatment accuracy and consistency, highlighting the need for improved treatment planning, real-time monitoring, and closed-loop control. Recent advances in image guidance and cavitation monitoring may improve this problem, but their routine integration remains limited, the main contradiction is that stronger mechanical effects may improve immune activation while simultaneously reducing spatial controllability and increasing off-target risk (174). Third, the therapeutic window has not been clearly defined. Although ultrasound can improve tissue permeability and stimulate immune responses, excessive exposure may also cause unwanted sterile inflammation, vascular injury, or damage to surrounding tissues. Future studies must therefore better resolve the dose-response relationship between acoustic input and immune outcome. Fourth, the timing and safety of combining ultrasound with existing immunotherapies still need to be optimized. Although ultrasound may improve drug delivery and immune priming, poorly timed combinations may increase toxicity or reduce therapeutic synergy. Accordingly, more rational scheduling and safety-oriented combination design will be required for clinical translation.

To overcome these barriers, future studies should move beyond descriptive proof-of-concept demonstrations and place greater emphasis on mechanism-based and precision-guided strategies. Key priorities include standardizing dosimetry and reporting methods to improve cross-study comparability, strengthening real-time monitoring and feedback control to enhance treatment precision, and integrating spatial multi-omics with imaging and functional biomarkers to better characterize response patterns across different tumor contexts. Such efforts should facilitate the identification of predictive biomarkers, improve patient stratification, and clarify how ultrasound-driven mechanical bioeffects can be translated into durable immune remodeling rather than nonspecific inflammation. Collectively, these advances are expected to support the safer, more rational, and more effective integration of ultrasound with cancer immunotherapy.

## References

[1] Kroemer G Chan TA Eggermont AMM Galluzzi L . Immunosurveillance in clinical cancer management. CA: A Cancer J For Clin. (2024) 74:187–202. doi: 10.3322/caac.21818. PMID: 37880100 PMC10939974

[2] Al Bakir M Reading JL Gamble S Rosenthal R Uddin I Rowan A . Clonal driver neoantigen loss under Egfr Tki and immune selection pressures. Nature. (2025) 639(8056):1052–9. doi: 10.1038/s41586-025-08586-y. PMID: 39972134 PMC11946900

[3] O'Sullivan D Sanin DE Pearce EJ Pearce EL . Metabolic interventions in the immune response to cancer. Nat Rev Immunol. (2019) 19:324–35. doi: 10.1038/s41577-019-0140-9. PMID: 30820043

[4] Kim SK Cho SW . The evasion mechanisms of cancer immunity and drug intervention in the tumor microenvironment. Front Pharmacol. (2022) 13:868695. doi: 10.3389/fphar.2022.868695. PMID: 35685630 PMC9171538

[5] Qiu ZW Zhong YT Lu ZM Yan N Kong RJ Huang JQ . Breaking physical barrier of fibrotic breast cancer for photodynamic immunotherapy by remodeling tumor extracellular matrix and reprogramming cancer-associated fibroblasts. ACS Nano. (2024) 18:9713–35. doi: 10.1021/acsnano.4c01499. PMID: 38507590

[6] Li C Qiu S Liu X Guo F Zhai J Li Z . Extracellular matrix-derived mechanical force governs breast cancer cell stemness and quiescence transition through integrin-Ddr signaling. Signal Transduct Target Ther. (2023) 8:247. doi: 10.1038/s41392-023-01453-0. PMID: 37369642 PMC10300038

[7] Mittelheisser V Gensbittel V Bonati L Li W Tang L Goetz JG . Evidence and therapeutic implications of biomechanically regulated immunosurveillance in cancer and other diseases. Nat Nanotechnol. (2024) 19:281–97. doi: 10.1038/s41565-023-01535-8. PMID: 38286876

[8] Nia HT Munn LL Jain RK . Physical traits of cancer. Science. (2020) 370(6516):eaaz0868. doi: 10.1126/science.aaz0868. PMID: 33122355 PMC8274378

[9] Dubinsky TJ Cuevas C Dighe MK Kolokythas O Hwang JH . High-intensity focused ultrasound: current potential and oncologic applications. AJR Am J Roentgenol. (2008) 190:191–9. doi: 10.2214/ajr.07.2671. PMID: 18094311

[10] O'Reilly MA . Exploiting the mechanical effects of ultrasound for noninvasive therapy. Science. (2024) 385:eadp7206. doi: 10.1126/science.adp7206. PMID: 39265013

[11] Du H Bartleson JM Butenko S Alonso V Liu WF Winer DA . Tuning immunity through tissue mechanotransduction. Nat Rev Immunol. (2023) 23:174–88. doi: 10.1038/s41577-022-00761-w. PMID: 35974148 PMC9379893

[12] Rix A Heinrichs H Porte C Leenaars C Bleich A Kiessling F . Ultrasound-induced immune responses in tumors: a systematic review and meta-analysis. J Control Release. (2024) 371:146–57. doi: 10.1016/j.jconrel.2024.05.030. PMID: 38777126

[13] Yang Y Cheng Y Cheng L . The emergence of cancer sono-immunotherapy. Trends Immunol. (2024) 45:549–63. doi: 10.1016/j.it.2024.06.001. PMID: 38910097

[14] Liang W Liang B Yan K Zhang G Zhuo J Cai Y . Low-intensity pulsed ultrasound: a physical stimulus with immunomodulatory and anti-inflammatory potential. Ann BioMed Eng. (2024) 52:1955–81. doi: 10.1007/s10439-024-03523-y. PMID: 38683473

[15] Liu S Zhang Y Liu Y Wang W Gao S Yuan W . Ultrasound-targeted microbubble destruction remodels tumour microenvironment to improve immunotherapeutic effect. Br J Cancer. (2023) 128:715–25. doi: 10.1038/s41416-022-02076-y. PMID: 36463323 PMC9977958

[16] Gargalionis AN Papavassiliou KA Papavassiliou AG . Mechanobiology of solid tumors. Biochim Biophys Acta Mol Basis Dis. (2022) 1868:166555. doi: 10.1016/j.bbadis.2022.166555. PMID: 36150659

[17] Khanmohammadi M Mirzaalikhan Y Baratchi S . Immunity in motion: the role of mechanics in macrophage biology. Cell Chem Biol. (2025) 32:1442–57. doi: 10.1016/j.chembiol.2025.11.004. PMID: 41371224

[18] Papavassiliou KA Basdra EK Papavassiliou AG . The emerging promise of tumour mechanobiology in cancer treatment. Eur J Cancer (Oxford England: 1990). (2023) 190:112938. doi: 10.1016/j.ejca.2023.112938. PMID: 37390803

[19] Zhou H Wang M Zhang Y Su Q Xie Z Chen X . Functions and clinical significance of mechanical tumor microenvironment: cancer cell sensing, mechanobiology and metastasis. Cancer Commun (London England). (2022) 42:374–400. doi: 10.1002/cac2.12294. PMID: 35470988 PMC9118059

[20] He X Zhong L Wang N Zhao B Wang Y Wu X . Gastric cancer actively remodels mechanical microenvironment to promote chemotherapy resistance via Mscs-mediated mitochondrial transfer. Advanced Sci (Weinheim Baden-Wurttemberg Germany). (2024) 11:e2404994. doi: 10.1002/advs.202404994. PMID: 39392399 PMC11653701

[21] Mai Z Lin Y Lin P Zhao X Cui L . Modulating extracellular matrix stiffness: a strategic approach to boost cancer immunotherapy. Cell Death Dis. (2024) 15:307. doi: 10.1038/s41419-024-06697-4. PMID: 38693104 PMC11063215

[22] Bigos KJ Quiles CG Lunj S Smith DJ Krause M Troost EG . Tumour response to hypoxia: understanding the hypoxic tumour microenvironment to improve treatment outcome in solid tumours. Front Oncol. (2024) 14:1331355. doi: 10.3389/fonc.2024.1331355. PMID: 38352889 PMC10861654

[23] Barbazán J Matic Vignjevic D . Cancer associated fibroblasts: is the force the path to the dark side? Curr Opin Cell Biol. (2019) 56:71–9. doi: 10.1016/j.ceb.2018.09.002. PMID: 30308331

[24] Micalet A Upadhyay A Javanmardi Y de Brito CG Moeendarbary E Cheema U . Patient-specific colorectal-cancer-associated fibroblasts modulate tumor microenvironment mechanics. iScience. (2024) 27:110060. doi: 10.1016/j.isci.2024.110060. PMID: 38883829 PMC11179580

[25] Liu HH Xu Y Li CJ Hsu SJ Lin XH Zhang R . An Scd1-dependent mechanoresponsive pathway promotes Hcc invasion and metastasis through lipid metabolic reprogramming. Mol Therapy: J Am Soc Gene Ther. (2022) 30:2554–67. doi: 10.1016/j.ymthe.2022.03.015. PMID: 35358687 PMC9263248

[26] Wang S Yuan X Yang Z Zhang X Xu Z Yang L . Matrix stiffness-dependent Pd-L2 deficiency improves Smyd3/Xct-mediated ferroptosis and the efficacy of anti-Pd-1 in Hcc. J Adv Res. (2025) 73:265–82. doi: 10.1016/j.jare.2024.08.021. PMID: 39159723 PMC12225897

[27] Zhang Y Ling L Murad R Maganti S Manceau A Hetrick HA . Macropinocytosis maintains Caf subtype identity under metabolic stress in pancreatic cancer. Cancer Cell. (2025) 43:1677–1696.e15. doi: 10.1016/j.ccell.2025.06.021. PMID: 40712568 PMC12313264

[28] Liu Y Yao X Zhao Y Fang D Shi L Yang L . Mechanotransduction in response to Ecm stiffening impairs Cgas immune signaling in tumor cells. Cell Rep. (2023) 42:113213. doi: 10.1016/j.celrep.2023.113213. PMID: 37804510

[29] Zheng Y Zhou R Cai J Yang N Wen Z Zhang Z . Matrix stiffness triggers lipid metabolic cross-talk between tumor and stromal cells to mediate bevacizumab resistance in colorectal cancer liver metastases. Cancer Res. (2023) 83:3577–92. doi: 10.1158/0008-5472.Can-23-0025. PMID: 37610655 PMC10618741

[30] Sun X Zhou Y Sun S Qiu S Peng M Gong H . Cancer cells sense solid stress to enhance metastasis by Ckap4 phase separation-mediated microtubule branching. Cell Discov. (2024) 10:114. doi: 10.1038/s41421-024-00737-1. PMID: 39528501 PMC11554681

[31] Jain RK Martin JD Stylianopoulos T . The role of mechanical forces in tumor growth and therapy. Annu Rev BioMed Eng. (2014) 16:321–46. doi: 10.1146/annurev-bioeng-071813-105259. PMID: 25014786 PMC4109025

[32] Zhang S Grifno G Passaro R Regan K Zheng S Hadzipasic M . Intravital measurements of solid stresses in tumours reveal length-scale and microenvironmentally dependent force transmission. Nat BioMed Eng. (2023) 7:1473–92. doi: 10.1038/s41551-023-01080-8. PMID: 37640900 PMC10836235

[33] Stylianopoulos T Martin JD Snuderl M Mpekris F Jain SR Jain RK . Coevolution of solid stress and interstitial fluid pressure in tumors during progression: implications for vascular collapse. Cancer Res. (2013) 73:3833–41. doi: 10.1158/0008-5472.Can-12-4521. PMID: 23633490 PMC3702668

[34] Cheng G Tse J Jain RK Munn LL . Micro-environmental mechanical stress controls tumor spheroid size and morphology by suppressing proliferation and inducing apoptosis in cancer cells. PloS One. (2009) 4:e4632. doi: 10.1371/journal.pone.0004632. PMID: 19247489 PMC2645686

[35] Nia HT Datta M Kumar AS Siri S Ferraro GB Chatterjee S . Solid stress estimations via intraoperative 3d navigation in patients with brain tumors. Clin Cancer Res. (2025) 31:3571–80. doi: 10.1158/1078-0432.Ccr-24-4159. PMID: 40495423 PMC12351280

[36] Cambria E Coughlin MF Floryan MA Offeddu GS Shelton SE Kamm RD . Linking cell mechanical memory and cancer metastasis. Nat Rev Cancer. (2024) 24:216–28. doi: 10.1038/s41568-023-00656-5. PMID: 38238471 PMC11146605

[37] Xinghua S Luyao Z Bo L Xiqiao F . The mechanical problems in tumor and tumor microenvironment. Adv Mech. (2018) 48:1808. doi: 10.6052/1000-0992-16-039

[38] Jain RK Baxter LT . Mechanisms of heterogeneous distribution of monoclonal antibodies and other macromolecules in tumors: significance of elevated interstitial pressure. Cancer Res. (1988) 48:7022–32. 3191477

[39] Chang SF Chang CA Lee DY Lee PL Yeh YM Yeh CR . Tumor cell cycle arrest induced by shear stress: roles of integrins and Smad. Proc Natl Acad Sci USA. (2008) 105:3927–32. doi: 10.1073/pnas.0712353105. PMID: 18310319 PMC2268796

[40] Hofmann M Guschel M Bernd A Bereiter-Hahn J Kaufmann R Tandi C . Lowering of tumor interstitial fluid pressure reduces tumor cell proliferation in a xenograft tumor model. Neoplasia (New York NY). (2006) 8:89–95. doi: 10.1593/neo.05469. PMID: 16611401 PMC1578509

[41] Shields JD Fleury ME Yong C Tomei AA Randolph GJ Swartz MA . Autologous chemotaxis as a mechanism of tumor cell homing to lymphatics via interstitial flow and autocrine Ccr7 signaling. Cancer Cell. (2007) 11:526–38. doi: 10.1016/j.ccr.2007.04.020. PMID: 17560334

[42] Huang Q Hu X He W Zhao Y Hao S Wu Q . Fluid shear stress and tumor metastasis. Am J Cancer Res. (2018) 8:763–77. doi: 10.1097/01.ju.0001191640.03217.b9.09. PMID: 29888101 PMC5992512

[43] Li A Zhang T Zhang X Xu Z Liu H Yuan M . Flexocatalytic reduction of tumor interstitial fluid/solid pressure for efficient nanodrug penetration. ACS Nano. (2024) 18(7):5344–57. doi: 10.1021/acsnano.3c09316. PMID: 38330150

[44] Provenzano PP Eliceiri KW Campbell JM Inman DR White JG Keely PJ . Collagen reorganization at the tumor-stromal interface facilitates local invasion. BMC Med. (2006) 4:38. doi: 10.1186/1741-7015-4-38. PMID: 17190588 PMC1781458

[45] Wolf K Te Lindert M Krause M Alexander S Te Riet J Willis AL . Physical limits of cell migration: control by Ecm space and nuclear deformation and tuning by proteolysis and traction force. J Cell Biol. (2013) 201:1069–84. doi: 10.1083/jcb.201210152. PMID: 23798731 PMC3691458

[46] Das S Jegadeesan JT Basu B . Gelatin methacryloyl (Gelma)-based biomaterial inks: process science for 3d/4d printing and current status. Biomacromolecules. (2024) 25:2156–221. doi: 10.1021/acs.biomac.3c01271. PMID: 38507816

[47] Ren T Sun L Zheng Y Jiang Y Guo Y Ma J . Mechanical forces and immune cells in the tumor microenvironment: from regulation mechanisms to therapeutic strategies. Int J Surg. (2025) 111:5420–34. doi: 10.1097/js9.0000000000002636. PMID: 40478969

[48] Zhang J Li J Hou Y Lin Y Zhao H Shi Y . Osr2 functions as a biomechanical checkpoint to aggravate Cd8(+) T cell exhaustion in tumor. Cell. (2024) 187:3409–3426.e24. doi: 10.1016/j.cell.2024.04.023. PMID: 38744281

[49] Peng DH Rodriguez BL Diao L Chen L Wang J Byers LA . Collagen promotes anti-Pd-1/Pd-L1 resistance in cancer through Lair1-dependent Cd8(+) T cell exhaustion. Nat Commun. (2020) 11:4520. doi: 10.1038/s41467-020-18298-8. PMID: 32908154 PMC7481212

[50] Lei K Kurum A Kaynak M Bonati L Han Y Cencen V . Cancer-cell stiffening via cholesterol depletion enhances adoptive T-cell immunotherapy. Nat BioMed Eng. (2021) 5:1411–25. doi: 10.1038/s41551-021-00826-6. PMID: 34873307 PMC7612108

[51] Luo Z Yao X Li M Fang D Fei Y Cheng Z . Modulating tumor physical microenvironment for fueling car-t cell therapy. Adv Drug Delivery Rev. (2022) 185:114301. doi: 10.1016/j.addr.2022.114301. PMID: 35439570

[52] Jones D Wang Z Chen IX Zhang S Banerji R Lei PJ . Solid stress impairs lymphocyte infiltration into lymph-node metastases. Nat BioMed Eng. (2021) 5:1426–36. doi: 10.1038/s41551-021-00766-1. PMID: 34282290 PMC8678215

[53] Alamán-Díez P Ferrer-Royo S Salafranca CO Compaired PM Balsas P Pardo J . Elevated interstitial fluid pressure promotes spheroid growth and reduces car-t therapeutic efficacy in solid tumors. Acta Biomater. (2026) 213:426–38. doi: 10.1016/j.actbio.2026.01.050. PMID: 41616888

[54] Ashby JF Schmidt J Kc N Kurum A Koch C Harari A . Microfluidic t cell selection by cellular avidity. Adv Healthc Mater. (2022) 11:e2200169. doi: 10.1002/adhm.202200169. PMID: 35657072 PMC11468699

[55] Robertson C Sebastian A Hinckley A Rios-Arce ND Hynes WF Edwards SA . Extracellular matrix modulates t cell clearance of Malignant cells *in vitro*. Biomaterials. (2022) 282:121378. doi: 10.1016/j.biomaterials.2022.121378. PMID: 35121359

[56] Su Y Yin X . The molecular mechanism of macrophages in response to mechanical stress. Ann BioMed Eng. (2025) 53:318–30. doi: 10.1007/s10439-024-03616-8. PMID: 39354279

[57] Yu KX Yuan WJ Wang HZ Li YX . Extracellular matrix stiffness and tumor-associated macrophage polarization: new fields affecting immune exclusion. Cancer Immunol Immunother. (2024) 73:115. doi: 10.1007/s00262-024-03675-9. PMID: 38693304 PMC11063025

[58] Xiong J Xiao R Zhao J Zhao Q Luo M Li F . Matrix stiffness affects tumor-associated macrophage functional polarization and its potential in tumor therapy. J Transl Med. (2024) 22:85. doi: 10.1186/s12967-023-04810-3. PMID: 38246995 PMC10800063

[59] Chen M Zhang Y Zhou P Liu X Zhao H Zhou X . Substrate stiffness modulates bone marrow-derived macrophage polarization through nf-κb signaling pathway. Bioact Mater. (2020) 5:880–90. doi: 10.1016/j.bioactmat.2020.05.004. PMID: 32637751 PMC7332470

[60] Salmaninejad A Valilou SF Soltani A Ahmadi S Abarghan YJ Rosengren RJ . Tumor-associated macrophages: role in cancer development and therapeutic implications. Cell Oncol (Dordr). (2019) 42:591–608. doi: 10.1007/s13402-019-00453-z. PMID: 31144271 PMC12994359

[61] Huang X Li Z Huang Y Zhang Q Cui Y Shi X . Vimentin intermediate filaments coordinate actin stress fibers and podosomes to determine the extracellular matrix degradation by macrophages. Dev Cell. (2025) 60(12):1669–85.e6. doi: 10.1016/j.devcel.2025.01.016. PMID: 39952241

[62] Dallavalasa S Beeraka NM Basavaraju CG Tulimilli SV Sadhu SP Rajesh K . The role of tumor associated macrophages (tams) in cancer progression, chemoresistance, angiogenesis and metastasis - current status. Curr Med Chem. (2021) 28:8203–36. doi: 10.2174/0929867328666210720143721. PMID: 34303328

[63] Zhu G Zhang R Xie Q Li P Wang F Wang L . Shish-kebab structure fiber with nano and micro diameter regulate macrophage polarization for anti-inflammatory and bone differentiation. Mater Today Bio. (2023) 23:100880. doi: 10.1016/j.mtbio.2023.100880. PMID: 38149017 PMC10750111

[64] Wong DCP Ding JL . The mechanobiology of nk cells- 'forcing nk to sense' target cells. Biochim Biophys Acta Rev Cancer. (2023) 1878:188860. doi: 10.1016/j.bbcan.2023.188860. PMID: 36791921

[65] Henke E Nandigama R Ergün S . Extracellular matrix in the tumor microenvironment and its impact on cancer therapy. Front Mol Biosci. (2019) 6:160. doi: 10.3389/fmolb.2019.00160. PMID: 32118030 PMC7025524

[66] Hu B Xin Y Hu G Li K Tan Y . Fluid shear stress enhances natural killer cell's cytotoxicity toward circulating tumor cells through nkg2d-mediated mechanosensing. APL Bioeng. (2023) 7:036108. doi: 10.1063/5.0156628. PMID: 37575881 PMC10423075

[67] Mennens SFB van den Dries K Cambi A . Role for mechanotransduction in macrophage and dendritic cell immunobiology. Results Probl Cell Differ. (2017) 62:209–42. doi: 10.1007/978-3-319-54090-0_9. PMID: 28455711

[68] Calmettes V Quintanilla MA Lacerda Mariano L Piel M Moreau HD Lennon-Duménil AM . Mechanosensing in dendritic cells. Immunol Rev. (2026) 337:e70086. doi: 10.1111/imr.70086. PMID: 41392598 PMC12703228

[69] Song MS Nam JH Noh KE Lim DS . Dendritic cell-based immunotherapy: the importance of dendritic cell migration. J Immunol Res. (2024) 2024:7827246. doi: 10.1155/2024/7827246. PMID: 38628676 PMC11019573

[70] Sapudom J Alatoom A Mohamed WKE Garcia-Sabaté A McBain I Nasser RA . Dendritic cell immune potency on 2d and in 3d collagen matrices. Biomater Sci. (2020) 8:5106–20. doi: 10.1039/d0bm01141j. PMID: 32812979

[71] Dombroski JA Rowland SJ Fabiano AR Knoblauch SV Hope JM King MR . Fluid shear stress enhances dendritic cell activation. Immunobiology. (2023) 228:152744. doi: 10.1016/j.imbio.2023.152744. PMID: 37729773 PMC10841200

[72] Quartey BC Torres G ElGindi M Alatoom A Sapudom J Teo JCM . Tug of war: understanding the dynamic interplay of tumor biomechanical environment on dendritic cell function. Mechanobiology Med. (2024) 2:100068. doi: 10.1016/j.mbm.2024.100068. PMID: 40395498 PMC12082323

[73] Sun BY Wang ZT Chen KZ Song Y Wu JF Zhang D . Mobilization and activation of tumor-infiltrating dendritic cells inhibits lymph node metastasis in intrahepatic cholangiocarcinoma. Cell Death Discov. (2024) 10:304. doi: 10.1038/s41420-024-02079-z. PMID: 38926350 PMC11208581

[74] Yuan Z Ye L Feng X Zhou T Zhou Y Zhu S . Yap-dependent induction of cd47-enriched extracellular vesicles inhibits dendritic cell activation and ameliorates hepatic ischemia-reperfusion injury. Oxid Med Cell Longev. (2021) 2021:6617345. doi: 10.1155/2021/6617345. PMID: 34239692 PMC8241504

[75] Baratchi S Danish H Chheang C Zhou Y Huang A Lai A . Piezo1 expression in neutrophils regulates shear-induced netosis. Nat Commun. (2024) 15:7023. doi: 10.1038/s41467-024-51211-1. PMID: 39174529 PMC11341855

[76] Scheiermann C Kunisaki Y Jang JE Frenette PS . Neutrophil microdomains: linking heterocellular interactions with vascular injury. Curr Opin Hematol. (2010) 17:25–30. doi: 10.1097/MOH.0b013e328333d2a3. PMID: 19923987 PMC3351007

[77] Agarwal S Loder SJ Cholok D Li J Bian G Yalavarthi S . Disruption of neutrophil extracellular traps (nets) links mechanical strain to post-traumatic inflammation. Front Immunol. (2019) 10:2148. doi: 10.3389/fimmu.2019.02148. PMID: 31708911 PMC6821718

[78] Jacobson EC Perry JK Long DS Olins AL Olins DE Wright BE . Migration through a small pore disrupts inactive chromatin organization in neutrophil-like cells. BMC Biol. (2018) 16:142. doi: 10.1186/s12915-018-0608-2. PMID: 30477489 PMC6257957

[79] Brunetti RM Kockelkoren G Raghavan P Bell GRR Britain D Puri N . Wasp integrates substrate topology and cell polarity to guide neutrophil migration. J Cell Biol. (2022) 221(2):e202104046. doi: 10.1083/jcb.202104046. PMID: 34964841 PMC8719638

[80] Shahzad MH Rayes RF Cools-Lartigue J Spicer JD . Neutrophil extracellular traps in cancer. Nat Rev Cancer. (2025) 26(2):104–17. doi: 10.1038/s41568-025-00888-7. PMID: 41224972

[81] Horaguchi S Nakahara Y Igarashi Y Kouro T Wei F Murotani K . Prognostic significance of plasma neutrophil extracellular trap levels in patients with non-small cell lung cancer treated with immune checkpoint inhibitors. Biomedicines. (2024) 12(8):1831. doi: 10.3390/biomedicines12081831. PMID: 39200295 PMC11351864

[82] Adams S Wuescher LM Worth R Yildirim-Ayan E . Mechano-immunomodulation: mechanoresponsive changes in macrophage activity and polarization. Ann BioMed Eng. (2019) 47:2213–31. doi: 10.1007/s10439-019-02302-4. PMID: 31218484 PMC7043232

[83] Lewis AH Cui AF McDonald MF Grandl J . Transduction of repetitive mechanical stimuli by piezo1 and piezo2 ion channels. Cell Rep. (2017) 19:2572–85. doi: 10.1016/j.celrep.2017.05.079. PMID: 28636944 PMC5646378

[84] Ambattu LA Yeo LY . Sonomechanobiology: vibrational stimulation of cells and its therapeutic implications. Biophys Rev (Melville). (2023) 4:021301. doi: 10.1063/5.0127122. PMID: 38504927 PMC10903386

[85] Ho YJ Li JP Fan CH Liu HL Yeh CK . Ultrasound in tumor immunotherapy: current status and future developments. J Control Release. (2020) 323:12–23. doi: 10.1016/j.jconrel.2020.04.023. PMID: 32302759

[86] Devos C Bampouli A Brozzi E Stefanidis GD Dusselier M Van Gerven T . Ultrasound mechanisms and their effect on solid synthesis and processing: a review. Chem Soc Rev. (2025) 54:85–115. doi: 10.1039/d4cs00148f. PMID: 39439231 PMC11496938

[87] Bucci F Lasiecka I . Feedback control of the acoustic pressure in ultrasonic wave propagation. Optimization. (2019) 68:1811–54. doi: 10.1080/02331934.2018.1504051. PMID: 37339054

[88] Shriki J . Ultrasound physics. Crit Care Clin. (2014) 30:1–24. doi: 10.1016/j.ccc.2013.08.004. PMID: 24295839

[89] Bergman E Goldbart R Traitel T Amar-Lewis E Zorea J Yegodayev K . Cell stiffness predicts cancer cell sensitivity to ultrasound as a selective superficial cancer therapy. Bioeng Transl Med. (2021) 6:e10226. doi: 10.1002/btm2.10226. PMID: 34589601 PMC8459597

[90] O'Brien WD . Ultrasound-biophysics mechanisms. Prog Biophys Mol Biol. (2007) 93:212–55. doi: 10.1016/j.pbiomolbio.2006.07.010. PMID: 16934858 PMC1995002

[91] Iacoponi F Cafarelli A Fontana F Pratellesi T Dumont E Barravecchia I . Optimal low-intensity pulsed ultrasound stimulation for promoting anti-inflammatory effects in macrophages. APL Bioeng. (2023) 7:016114. doi: 10.1063/5.0137881. PMID: 36968453 PMC10036142

[92] Hsu CH Pan YJ Zheng YT Lo RY Yang FY . Ultrasound reduces inflammation by modulating m1/m2 polarization of microglia through stat1/stat6/pparγ signaling pathways. CNS Neurosci Ther. (2023) 29:4113–23. doi: 10.1111/cns.14333. PMID: 37401041 PMC10651950

[93] Wang S Hossack JA Klibanov AL Mauldin FW . Binding dynamics of targeted microbubbles in response to modulated acoustic radiation force. Phys Med Biol. (2014) 59:465–84. doi: 10.1088/0031-9155/59/2/465. PMID: 24374866 PMC4068277

[94] He Y Feng Y Qiu D Lin M Jin H Hu Z . Regulation of ifp in solid tumours through acoustic pressure to enhance infiltration of nanoparticles of various sizes. J Drug Target. (2024) 32:964–76. doi: 10.1080/1061186x.2024.2367579. PMID: 38884143

[95] Chen H Brayman AA Bailey MR Matula TJ . Blood vessel rupture by cavitation. Urol Res. (2010) 38:321–6. doi: 10.1007/s00240-010-0302-5. PMID: 20680255 PMC3192534

[96] Guerassimoff L De Smedt SC Sauvage F Baudoin M . Acoustic tweezers for targeted drug delivery. Adv Drug Delivery Rev. (2025) 220:115551. doi: 10.1016/j.addr.2025.115551. PMID: 39988259

[97] Yang Y Li Q Guo X Tu J Zhang D . Mechanisms underlying sonoporation: interaction between microbubbles and cells. Ultrason Sonochem. (2020) 67:105096. doi: 10.1016/j.ultsonch.2020.105096. PMID: 32278246

[98] Cirincione R Di Maggio FM Forte GI Minafra L Bravatà V Castiglia L . High-intensity focused ultrasound- and radiation therapy-induced immuno-modulation: comparison and potential opportunities. Ultrasound Med Biol. (2017) 43:398–411. doi: 10.1016/j.ultrasmedbio.2016.09.020. PMID: 27780661

[99] Bouakaz A Michel Escoffre J . From concept to early clinical trials: 30 years of microbubble-based ultrasound-mediated drug delivery research. Adv Drug Delivery Rev. (2024) 206:115199. doi: 10.1016/j.addr.2024.115199. PMID: 38325561

[100] Lentacker I De Cock I Deckers R De Smedt SC Moonen CT . Understanding ultrasound induced sonoporation: definitions and underlying mechanisms. Adv Drug Delivery Rev. (2014) 72:49–64. doi: 10.1016/j.addr.2013.11.008. PMID: 24270006

[101] VanBavel E . Effects of shear stress on endothelial cells: Possible relevance for ultrasound applications. Prog Biophys Mol Biol. (2007) 93:374–83. doi: 10.1016/j.pbiomolbio.2006.07.017. PMID: 16970981

[102] Parsons JE Cain CA Abrams GD Fowlkes JB . Pulsed cavitational ultrasound therapy for controlled tissue homogenization. Ultrasound Med Biol. (2006) 32:115–29. doi: 10.1016/j.ultrasmedbio.2005.09.005. PMID: 16364803

[103] McGinnis R Song B Kim H Lorenzon A Shi J Zhao L . Histotripsy dose impacts treated tumor immune infiltration and survival outcomes in a murine B16f10 melanoma model. Cancers (Basel). (2025) 17(23):3773. doi: 10.3390/cancers17233773. PMID: 41374975 PMC12691148

[104] Guo Y Lee H Kim C Park C Yamamichi A Chuntova P . Ultrasound frequency-controlled microbubble dynamics in brain vessels regulate the enrichment of inflammatory pathways in the blood-brain barrier. Nat Commun. (2024) 15:8021. doi: 10.1038/s41467-024-52329-y. PMID: 39271721 PMC11399249

[105] Baez A Singh D He S Hajiaghayi M Gholizadeh F Darlington PJ . Immunomodulation of human T cells by microbubble-mediated focused ultrasound. Front Immunol. (2024) 15:1486744. doi: 10.3389/fimmu.2024.1486744. PMID: 39502696 PMC11534865

[106] Lafond M Payne A Lafon C . Therapeutic ultrasound transducer technology and monitoring techniques: A review with clinical examples. Int J Hyperthermia. (2024) 41:2389288. doi: 10.1080/02656736.2024.2389288. PMID: 39134055 PMC11375802

[107] Xu Z Khokhlova TD Cho CS Khokhlova VA . Histotripsy: A method for mechanical tissue ablation with ultrasound. Annu Rev BioMed Eng. (2024) 26:141–67. doi: 10.1146/annurev-bioeng-073123-022334. PMID: 38346277 PMC11837764

[108] American Institute of Ultrasound in Medicine . Statement on Biological Effects of Therapeutic Ultrasound. (2023). Available online at: https://www.aium.org/resources/official-statements/view/statement-on-biological-effects-of-therapeutic-ultrasound (Accessed May 23, 2026). 10.1002/jum.16315 37584480

[109] Aium Official Statement . Statement on biological effects of therapeutic ultrasound. J Ultrasound Med. (2023) 42:E68–e73. doi: 10.1002/jum.16315. PMID: 37584480

[110] Pan Y Yoon S Sun J Huang Z Lee C Allen M . Mechanogenetics for the remote and noninvasive control of cancer immunotherapy. Proc Natl Acad Sci USA. (2018) 115:992–7. doi: 10.1073/pnas.1714900115. PMID: 29343642 PMC5798350

[111] Yang R Zhang X Zhang Y Man Y Yang X . Low-intensity pulsed ultrasound promotes a Treg-like phenotype and suppresses a Th17-like phenotype in Cd4(+) T cells via Yap/Taz activation *in vitro*. J Inflammation Res. (2025) 18:13593–608. doi: 10.2147/jir.S548291. PMID: 41059283 PMC12499271

[112] Yin J Bao Y Xu M Li P Zhang Z Xue H . Anti-inflammatory role of low-intensity pulsed ultrasound in inhibiting lipopolysaccharide-induced M1 polarization of Raw264.7 cells via Wnt2b/Axin/B-catenin. PeerJ. (2024) 12:e18448. doi: 10.7717/peerj.18448. PMID: 39553710 PMC11568821

[113] Pu Y Zhou B Bing J Wang L Chen M Shen Y . Ultrasound-triggered and glycosylation inhibition-enhanced tumor piezocatalytic immunotherapy. Nat Commun. (2024) 15:9023. doi: 10.1038/s41467-024-53392-1. PMID: 39424801 PMC11489718

[114] Xu S Meng L Hu Q Li F Zhang J Kong N . Closed-loop control of macrophage engineering enabled by focused-ultrasound responsive mechanoluminescence nanoplatform for precise cancer immunotherapy. Small. (2024) 20:e2401398. doi: 10.1002/smll.202401398. PMID: 39101277

[115] Hong X Rzeczycki PM Keswani RK Murashov MD Fan Z Deng CX . Acoustic tweezing cytometry for mechanical phenotyping of macrophages and mechanopharmaceutical cytotripsy. Sci Rep. (2019) 9:5702. doi: 10.1038/s41598-019-42180-3. PMID: 30952950 PMC6450871

[116] Liang X Guo Z Chen Z . Advances in macrophage mechanoimmunology: Understanding the role of ultrasound mechanical effect. Ultrasound Med Biol. (2026) 52:259–71. doi: 10.1016/j.ultrasmedbio.2025.10.010. PMID: 41219090

[117] Kim M Lee S Ki CS . Cellular behavior of Raw264.7 cells in 3d poly(ethylene glycol) hydrogel niches. ACS Biomater Sci Eng. (2019) 5:922–32. doi: 10.1021/acsbiomaterials.8b01150. PMID: 33405849

[118] Feng R Gu A Zengin G Du M . Low-intensity pulsed ultrasound enhances Nk cell adoptive therapy by modulating the Wnt/B-catenin signaling pathway. Bio Integration. (2025) 6:1–8. doi: 10.15212/bioi-2025-0036

[119] Zhang R Yang W Zhou Z Ding M Wang H Yuan W . Ultrasound-activated piezoelectric nanoparticles targeting and activating Nk cells for tumor immunotherapy. Adv Mater. (2025) 37:e08101. doi: 10.1002/adma.202508101. PMID: 40916189

[120] Dewitte H Van Lint S Heirman C Thielemans K De Smedt SC Breckpot K . The potential of antigen and Trimix sonoporation using Mrna-loaded microbubbles for ultrasound-triggered cancer immunotherapy. J Control Release. (2014) 194:28–36. doi: 10.1016/j.jconrel.2014.08.011. PMID: 25151979

[121] Jahangiri S Bourdages S Skora E Stagg J Yu F . Atp released by ultrasound targeted microbubble cavitation induces vascular inflammation and improves immune checkpoint blockade efficacy. Theranostics. (2025) 15:5220–37. doi: 10.7150/thno.105857. PMID: 40303330 PMC12036883

[122] Korosoglou G Hardt SE Bekeredjian R Jenne J Konstantin M Hagenmueller M . Ultrasound exposure can increase the membrane permeability of human neutrophil granulocytes containing microbubbles without causing complete cell destruction. Ultrasound Med Biol. (2006) 32:297–303. doi: 10.1016/j.ultrasmedbio.2005.11.010. PMID: 16464675

[123] Ireland AS Oliver TG . Neutrophils create an impenetrable shield between tumor and cytotoxic immune cells. Immunity. (2020) 52:729–31. doi: 10.1016/j.immuni.2020.04.009. PMID: 32433945 PMC7851833

[124] Korosoglou G da Silva KG Hansen A Hardt S Browatzki M Kranzhoefer R . Ultrasound contrast agents can influence the respiratory burst activity of human neutrophil granulocytes. Ultrasound Med Biol. (2004) 30:75–81. doi: 10.1016/j.ultrasmedbio.2003.09.008. PMID: 14962611

[125] Joiner JB Pylayeva-Gupta Y Dayton PA . Focused ultrasound for immunomodulation of the tumor microenvironment. J Immunol. (2020) 205:2327–41. doi: 10.4049/jimmunol.1901430. PMID: 33077668 PMC7583653

[126] Wang Y Liu C Lyu R Zhang Y . Application and prospects of ultrasound combined with immunotherapy in cancer treatment of intensive care. Front Immunol. (2025) 16:1670527. doi: 10.3389/fimmu.2025.1670527. PMID: 41394807 PMC12696427

[127] Arrieta VA Gould A Kim KS Habashy KJ Dmello C Vázquez-Cervantes GI . Ultrasound-mediated delivery of doxorubicin to the brain results in immune modulation and improved responses to Pd-1 blockade in gliomas. Nat Commun. (2024) 15:4698. doi: 10.1038/s41467-024-48326-w. PMID: 38844770 PMC11156895

[128] Feng X Cao F Wu X Xie W Wang P Jiang H . Targeting extracellular matrix stiffness for cancer therapy. Front Immunol. (2024) 15:1467602. doi: 10.3389/fimmu.2024.1467602. PMID: 39697341 PMC11653020

[129] Pallumeera M Hong M Giang JC Makary MS . Histotripsy: Recent advances, clinical applications, and future prospects. Cancers (Basel). (2025) 17(18):3072. doi: 10.3390/cancers17183072. PMID: 41008913 PMC12469116

[130] van den Bijgaart RJE Mekers VE Schuurmans F Raaijmakers TK Wassink M Veltien A . Mechanical high-intensity focused ultrasound creates unique tumor debris enhancing dendritic cell-induced T cell activation. Front Immunol. (2022) 13:1038347. doi: 10.3389/fimmu.2022.1038347. PMID: 36569907 PMC9768443

[131] Bader KB Vlaisavljevich E Maxwell AD . For whom the bubble grows: Physical principles of bubble nucleation and dynamics in histotripsy ultrasound therapy. Ultrasound Med Biol. (2019) 45:1056–80. doi: 10.1016/j.ultrasmedbio.2018.10.035. PMID: 30922619 PMC6524960

[132] Hoogenboom M Eikelenboom D den Brok MH Heerschap A Fütterer JJ Adema GJ . Mechanical high-intensity focused ultrasound destruction of soft tissue: Working mechanisms and physiologic effects. Ultrasound Med Biol. (2015) 41:1500–17. doi: 10.1016/j.ultrasmedbio.2015.02.006. PMID: 25813532

[133] Abe S Nagata H Crosby EJ Inoue Y Kaneko K Liu CX . Combination of ultrasound-based mechanical disruption of tumor with immune checkpoint blockade modifies tumor microenvironment and augments systemic antitumor immunity. J Immunother Cancer. (2022) 10:e003717. doi: 10.1136/jitc-2021-003717. PMID: 35039461 PMC8765068

[134] Lee NS Yoon CW Wang Q Moon S Koo KM Jung H . Focused ultrasound stimulates Er localized mechanosensitive Pannexin-1 to mediate intracellular calcium release in invasive cancer cells. Front Cell Dev Biol. (2020) 8:504. doi: 10.3389/fcell.2020.00504. PMID: 32656213 PMC7325310

[135] Thim EA Kitelinger LE Rivera-Escalera F Mathew AS Elliott MR Bullock TNJ . Focused ultrasound ablation of melanoma with boiling histotripsy yields abscopal tumor control and antigen-dependent dendritic cell activation. Theranostics. (2024) 14:1647–61. doi: 10.7150/thno.92089. PMID: 38389838 PMC10879863

[136] Padilla F Foley J Timbie K Bullock TNJ Sheybani ND . Guidelines for immunological analyses following focused ultrasound treatment. J Immunother Cancer. (2023) 11(11):e007455. doi: 10.1136/jitc-2023-007455. PMID: 38007236 PMC10679984

[137] Zheng T Yu X Yu C Xu W Fan Z Zhou Y . Tumor-associated endothelial cells in tumor immune escape and immunotherapy: Multifaceted roles and treatment approaches. biomark Res. (2025) 14:10. doi: 10.1186/s40364-025-00883-y. PMID: 41444687 PMC12784535

[138] Wang G Zhang C Jiang Y Song Y Chen J Sun Y . Ultrasonic cavitation‐assisted and acid‐activated transcytosis of liposomes for universal active tumor penetration. Adv Funct Mater. (2021) 31(34):2102786. doi: 10.1002/adfm.202102786. PMID: 41531421

[139] Meijlink B van der Kooij HR Wang Y Li H Huveneers S Kooiman K . Ultrasound-activated microbubbles mediate F-actin disruptions and endothelial gap formation during sonoporation. J Control Release. (2024) 376:1176–89. doi: 10.1016/j.jconrel.2024.10.066. PMID: 39500409

[140] Chien CY Xu L Yuan J Fadera S Stark AH Athiraman U . Quality assurance for focused ultrasound-induced blood-brain barrier opening procedure using passive acoustic detection. EBioMedicine. (2024) 102:105066. doi: 10.1016/j.ebiom.2024.105066. PMID: 38531173 PMC10987799

[141] Koutsi M Stylianopoulos T Mpekris F . Optimizing therapeutic outcomes with mechanotherapy and ultrasound sonopermeation in solid tumors. PloS Comput Biol. (2025) 21:e1012676. doi: 10.1371/journal.pcbi.1012676. PMID: 40986621 PMC12483211

[142] Li N Tang J Yang J Zhu B Wang X Luo Y . Tumor perfusion enhancement by ultrasound stimulated microbubbles potentiates Pd-L1 blockade of Mc38 colon cancer in mice. Cancer Lett. (2021) 498:121–9. doi: 10.1016/j.canlet.2020.10.046. PMID: 33129956

[143] Fan CH Ting CY Liu HL Huang CY Hsieh HY Yen TC . Antiangiogenic-targeting drug-loaded microbubbles combined with focused ultrasound for glioma treatment. Biomaterials. (2013) 34:2142–55. doi: 10.1016/j.biomaterials.2012.11.048. PMID: 23246066

[144] Liu J You Q Liang F Ma L Zhu L Wang C . Ultrasound-nanovesicles interplay for theranostics. Adv Drug Delivery Rev. (2024) 205:115176. doi: 10.1016/j.addr.2023.115176. PMID: 38199256

[145] Wu N Zhang Q Tang R Deng L Cao Y Fu B . Ultrasound visualization of spatiotemporal autophagy-regulated nanodroplets for amplifying Icb in melanoma via remodeling tumor inflammatory microenvironment. ACS Appl Mater Interfaces. (2025) 17:29364–78. doi: 10.1021/acsami.5c03394. PMID: 40331917 PMC12100593

[146] Yang C Du M Yan F Chen Z . Focused ultrasound improves Nk-92mi cells infiltration into tumors. Front Pharmacol. (2019) 10:326. doi: 10.3389/fphar.2019.00326. PMID: 31057396 PMC6482214

[147] Poon C Pellow C Hynynen K . Neutrophil recruitment and leukocyte response following focused ultrasound and microbubble mediated blood-brain barrier treatments. Theranostics. (2021) 11:1655–71. doi: 10.7150/thno.52710. PMID: 33408773 PMC7778596

[148] Wang X Li F Zhang J Guo L Shang M Sun X . A combination of Pd-L1-targeted Il-15 Mrna nanotherapy and ultrasound-targeted microbubble destruction for tumor immunotherapy. J Control Release. (2024) 367:45–60. doi: 10.1016/j.jconrel.2024.01.039. PMID: 38246204

[149] Fang M Zheng J Song Q Huang J Cao R Li P . Breaking apoptosis-induced immune silence: Ultrasound-activated nano-oncolytic therapy reinvigorates antitumor immunity. Adv Mater. (2025) 37:e2508681. doi: 10.1002/adma.202508681. PMID: 40525766

[150] Li Z Zhang B Duan S Liu R Wang Y Wang Y . Ultrasound-activated nanovesicles for adenosine exhaustion and immune checkpoint blockade in cancer immunotherapy. J Control Release. (2025) 385:113988. doi: 10.1016/j.jconrel.2025.113988. PMID: 40582643

[151] McMahon D O'Reilly MA Hynynen K . Therapeutic agent delivery across the blood-brain barrier using focused ultrasound. Annu Rev BioMed Eng. (2021) 23:89–113. doi: 10.1146/annurev-bioeng-062117-121238. PMID: 33752471 PMC11979953

[152] Sonabend AM Gould A Amidei C Ward R Schmidt KA Zhang DY . Repeated blood-brain barrier opening with an implantable ultrasound device for delivery of albumin-bound paclitaxel in patients with recurrent glioblastoma: a phase 1 trial. Lancet Oncol. (2023) 24:509–22. doi: 10.1016/s1470-2045(23)00112-2. PMID: 37142373 PMC10256454

[153] Chen Z Sang L Qixi Z Li X Liu Y Bai Z . Ultrasound-responsive nanoparticles for imaging and therapy of brain tumors. Mater Today Bio. (2025) 32:101661. doi: 10.1016/j.mtbio.2025.101661. PMID: 40206140 PMC11979416

[154] Li X Khorsandi S Wang Y Santelli J Huntoon K Nguyen N . Cancer immunotherapy based on image-guided sting activation by nucleotide nanocomplex-decorated ultrasound microbubbles. Nat Nanotechnol. (2022) 17:891–9. doi: 10.1038/s41565-022-01134-z. PMID: 35637356 PMC9378430

[155] Yin H Hu X Xie C Li Y Gao Y Zeng H . A T-cell inspired sonoporation system enhances low-dose X-ray-mediated pyroptosis and radioimmunotherapy efficacy by restoring gasdermin-E expression. Adv Mater. (2024) 36:e2401384. doi: 10.1002/adma.202401384. PMID: 38521987

[156] Li H Zhang Y Shu H Lv W Su C Nie F . Highlights in ultrasound-targeted microbubble destruction-mediated gene/drug delivery strategy for treatment of Malignancies. Int J Pharm. (2022) 613:121412. doi: 10.1016/j.ijpharm.2021.121412. PMID: 34942327

[157] Einen C Snipstad S Wesche HF Nordlund V Devold EJ Amini N . Impact of the tumor microenvironment on delivery of nanomedicine in tumors treated with ultrasound and microbubbles. J Control Release. (2025) 378:656–70. doi: 10.1016/j.jconrel.2024.12.037. PMID: 39701458

[158] Sun S Wang P Sun S Liang X . Applications of micro/nanotechnology in ultrasound-based drug delivery and therapy for tumor. Curr Med Chem. (2021) 28:525–47. doi: 10.2174/0929867327666200212100257. PMID: 32048951

[159] Zhang J Zhang Y Cai Z Wei J Li H Li P . Augmentation of tumour perfusion by ultrasound and microbubbles: a preclinical study. Ultrasonics. (2024) 138:107219. doi: 10.1016/j.ultras.2023.107219. PMID: 38104380

[160] Bai L Luo T Tang J Zhang J Tan X Tang J . Ultrasound-induced tumor perfusion changes and doxorubicin delivery: a study on pulse length and pulse repetition frequency. J Ultrasound Med. (2024) 43:253–63. doi: 10.1002/jum.16355. PMID: 37853950

[161] Feng Y Qiu D He Y Jin H Chen L Xi F . Effect of ultrasound combined with microbubbles therapy on tumor hypoxic microenvironment. Front Pharmacol. (2024) 15:1502349. doi: 10.3389/fphar.2024.1502349. PMID: 39872052 PMC11769831

[162] Drzał A Delalande A Dziurman G Fournié M Pichon C Elas M . Increasing oxygen tension in tumor tissue using ultrasound sensitive O(2) microbubbles. Free Radic Biol Med. (2022) 193:567–78. doi: 10.1016/j.freeradbiomed.2022.11.005. PMID: 36356713

[163] Luo T Yao L Wu Y Zhang Y Lu L He P . Ultrasound-stimulated microbubbles cavitation combined with nitric oxide signaling pathway to alleviate tumor hypoperfusion and hypoxia in Mc38 tumor model. Acad Radiol. (2025) 32:4121–33. doi: 10.1016/j.acra.2025.03.034. PMID: 40234163

[164] Qiu D He Y Feng Y Lin M Lin Z Zhang Z . Tumor perfusion enhancement by microbubbles ultrasonic cavitation reduces tumor glycolysis metabolism and alleviate tumor acidosis. Front Oncol. (2024) 14:1424824. doi: 10.3389/fonc.2024.1424824. PMID: 39091919 PMC11291205

[165] Tijore A Margadant F Dwivedi N Morgan L Yao M Hariharan A . Ultrasound-mediated mechanical forces activate selective tumor cell apoptosis. Bioeng Transl Med. (2025) 10:e10737. doi: 10.1002/btm2.10737. PMID: 40060768 PMC11883105

[166] Wu SY Wang CH Kang ST Yu CF Chen FH Chiang CS . Power-Doppler-based Nh002 microbubble sonoporation with chemotherapy relieves hypoxia and enhances the efficacy of chemotherapy and immunotherapy for pancreatic tumors. Sci Rep. (2024) 14:8532. doi: 10.1038/s41598-024-54432-y. PMID: 38830912 PMC11148017

[167] Song B Queen H Ferris SF McGinnis R Karanam C Gatteno N . Histotripsy-focused ultrasound treatment abrogates tumor hypoxia responses and stimulates antitumor immune responses in melanoma. Mol Cancer Ther. (2025) 24:1088–98. doi: 10.1158/1535-7163.Mct-24-0715. PMID: 40105625 PMC12221799

[168] Martinez PJ Song JJ Garay FG Song KH Mufford T Steiner J . Comprehensive assessment of blood-brain barrier opening and sterile inflammatory response: unraveling the therapeutic window. Sci Rep. (2024) 14:17036. doi: 10.1038/s41598-024-67916-8. PMID: 39043894 PMC11266505

[169] Mouratidis P Ferreira RC Anbalagan S Chauhan R Rivens I Ter Haar G . Transcriptomic profiling of the immune response in orthotopic pancreatic tumours exposed to combined boiling histotripsy and oncolytic reovirus treatment. Pharmaceutics. (2025) 17(8):949. doi: 10.3390/pharmaceutics17080949. PMID: 40870972 PMC12389550

[170] Xu R Su X Qin X Liu Y Wu J Li X . An omics-based drug-Hifu combination therapy discovery for ferroptosis treatment of Tnbc. Biomaterials. (2026) 324:123535. doi: 10.1016/j.biomaterials.2025.123535. PMID: 40627961

[171] Wen PN Lin MS Chen JC . Mechanobiological ultrasound simulation reveals suppression of epithelial-to-mesenchymal transition and stemness programs in colorectal cancer. Cancer Biother Radiopharm. (2026), 10849785261422983. doi: 10.1177/10849785261422983. PMID: 41742663

[172] Feng W He P Wang Z Li W . Breakthroughs of ultrasound-targeted microbubble destruction in treating myocardial ischemia-reperfusion injury: from angiogenesis regulation to precise inflammation suppression. Drug Delivery. (2025) 32:2594555. doi: 10.1080/10717544.2025.2594555. PMID: 41369269 PMC12697267

[173] Wang Y Zhang H Xu Z Zhu W Chang S Wei J . Icariin-loaded Gelma hydrogel encapsulated potassium sodium niobate biomimetic piezoelectric scaffold regulates macrophage polarization to accelerate bone defect repair. Mater Today Bio. (2025) 35:102476. doi: 10.1016/j.mtbio.2025.102476. PMID: 41281663 PMC12639499

[174] Engelen Y Krysko DV Effimova I Breckpot K Versluis M De Smedt S . Optimizing high-intensity focused ultrasound-induced immunogenic cell-death using passive cavitation mapping as a monitoring tool. J Controlled Release. (2024) 375:389–403. doi: 10.1016/j.jconrel.2024.09.016. PMID: 39293525

[175] Zheng J Huang J Zhang L Wang M Xu L Dou X . Drug-loaded microbubble delivery system to enhance Pd-L1 blockade immunotherapy with remodeling immune microenvironment. Biomater Res. (2023) 27:9. doi: 10.1186/s40824-023-00350-5. PMID: 36759928 PMC9909878

[176] Zhao Y Shi D Guo L Shang M Sun X Meng D . Ultrasound targeted microbubble destruction-triggered nitric oxide release via nanoscale ultrasound contrast agent for sensitizing chemoimmunotherapy. J Nanobiotechnology. (2023) 21:35. doi: 10.1186/s12951-023-01776-8. PMID: 36717899 PMC9885630

[177] Tang Y Shen Q Lin P Chen Z Fan D Zhuo M . Apd-L1-facilitated theranostic and tumor microenvironment remodeling of pancreatic cancer via docetaxel-loaded phase-transformation nanoparticles triggered by low-intensity pulsed ultrasound. J Nanobiotechnology. (2025) 23:48. doi: 10.1186/s12951-025-03105-7. PMID: 39871305 PMC11773723

[178] Lan M Zhu L Wang Y Shen D Fang K Liu Y . Multifunctional nanobubbles carrying indocyanine green and paclitaxel for molecular imaging and the treatment of prostate cancer. J Nanobiotechnology. (2020) 18:121. doi: 10.1186/s12951-020-00650-1. PMID: 32883330 PMC7469305

[179] Hong D Yang J Guo J Zhang Y Chen Z . Ultrasound-targeted microbubble destruction enhances inhibitory effect of apatinib on angiogenesis in triple negative breast carcinoma xenografts. Anal Cell Pathol (Amst). (2021) 2021:8837950. doi: 10.1155/2021/8837950. PMID: 33959473 PMC8075700

[180] Huang J Zhang L Zheng J Lin Y Leng X Wang C . Microbubbles-assisted ultrasonication to promote tumor accumulation of therapeutics and modulation of tumor microenvironment for enhanced cancer treatments. Biomaterials. (2023) 299:122181. doi: 10.1016/j.biomaterials.2023.122181. PMID: 37276797

[181] Wu N Cao Y Liu Y Zhou Y He H Tang R . Low-intensity focused ultrasound targeted microbubble destruction reduces tumor blood supply and sensitizes anti-Pd-L1 immunotherapy. Front Bioeng Biotechnol. (2023) 11:1173381. doi: 10.3389/fbioe.2023.1173381. PMID: 37139047 PMC10150078

[182] Xie L Wang J Song L Jiang T Yan F . Cell-cycle dependent nuclear gene delivery enhances the effects of E-cadherin against tumor invasion and metastasis. Signal Transduction Targeted Ther. (2023) 8:182. doi: 10.1038/s41392-023-01398-4. PMID: 37150786 PMC10164743

[183] Liu Y Long T Zhang N Qiao B Yang Q Luo Y . Ultrasound-mediated long-circulating nanopolymer delivery of therapeutic Sirna and antisense micrornas leads to enhanced paclitaxel sensitivity in epithelial ovarian cancer chemotherapy. ACS Biomater Sci Eng. (2020) 6:4036–50. doi: 10.1021/acsbiomaterials.0c00330. PMID: 33463352

[184] Kida H Nishimura K Ogawa K Watanabe A Feril LB Irie Y . Nanobubble mediated gene delivery in conjunction with a hand-held ultrasound scanner. Front Pharmacol. (2020) 11:363. doi: 10.3389/fphar.2020.00363. PMID: 32300298 PMC7145407

[185] Sun W Ji P Zhou T Li Z Xing C Zhang L . Ultrasound responsive nanovaccine armed with engineered cancer cell membrane and Rna to prevent foreseeable metastasis. Adv Sci (Weinh). (2023) 10:e2301107. doi: 10.1002/advs.202301107. PMID: 37097746 PMC10323640

[186] Jugniot N Dahl JJ Paulmurugan R . Immunotheranostic microbubbles (Imbs) - a modular platform for dendritic cell vaccine delivery applied to breast cancer immunotherapy. J Exp Clin Cancer Res. (2022) 41:299. doi: 10.1186/s13046-022-02501-3. PMID: 36224614 PMC9555090

[187] Singh MP Sethuraman SN Miller C Malayer J Ranjan A . Boiling histotripsy and in-situ Cd40 stimulation improve the checkpoint blockade therapy of poorly immunogenic tumors. Theranostics. (2021) 11:540–54. doi: 10.7150/thno.49517. PMID: 33391491 PMC7738858

[188] Pepple AL Guy JL McGinnis R Felsted AE Song B Hubbard R . Spatiotemporal local and abscopal cell death and immune responses to histotripsy focused ultrasound tumor ablation. Front Immunol. (2023) 14:1012799. doi: 10.3389/fimmu.2023.1012799. PMID: 36756111 PMC9900174

[189] Xiong X Zhou H Xu X Fu Q Wan Y Cao Y . Ultrasound molecular imaging enhances high-intensity focused ultrasound ablation on liver cancer with B7-H3-targeted microbubbles. Cancer Med. (2024) 13:e70341. doi: 10.1002/cam4.70341. PMID: 39431644 PMC11492419

[190] Yang J Liao M Wu Z Liu X Zheng Z Wang W . Perfluorohexane nanodroplet-assisted mechanical high intensity focused ultrasound cavitation: a strategy for hepatocellular carcinoma treatment. Acta Biomater. (2025) 195:297–308. doi: 10.1016/j.actbio.2025.01.061. PMID: 39894325

[191] Lee J Um W Moon H Joo H Song Y Park M . Evading doxorubicin-induced systemic immunosuppression using ultrasound-responsive liposomes combined with focused ultrasound. Pharmaceutics. (2022) 14(12):2603. doi: 10.3390/pharmaceutics14122603. PMID: 36559097 PMC9784431

[192] Qi T Jing Y Deng J Chang J Sun W Yang R . Boiling histotripsy using dual-frequency protocol on murine breast tumor model and promotes immune activation. IEEE Trans Ultrason Ferroelectr Freq Control. (2023) 70:1773–85. doi: 10.1109/tuffc.2023.3326561. PMID: 37871099

[193] Yu X Li X Chen Q Wang S Xu R He Y . High intensity focused ultrasound-driven nanomotor for effective ferroptosis-immunotherapy of Tnbc. Adv Sci (Weinh). (2024) 11:e2305546. doi: 10.1002/advs.202305546. PMID: 38342612 PMC11022700

[194] Glickstein B Bismuth M Gattegno R Bercovici T Shaul O Aronovich R . Volumetric nanodroplet-enhanced ultrasound surgery combined with immune checkpoint inhibition as a cancer therapy platform. Small. (2025) 21:e2411474. doi: 10.1002/smll.202411474. PMID: 40059532 PMC12160685

[195] Mouratidis PXE Costa M Rivens I Repasky EE Ter Haar G . Pulsed focused ultrasound can improve the anti-cancer effects of immune checkpoint inhibitors in murine pancreatic cancer. J R Soc Interface. (2021) 18:20210266. doi: 10.1098/rsif.2021.0266. PMID: 34229458 PMC8261215

[196] Yao Y Zheng Y Wu M Gao Y Yu Q Liu M . Cd133-targeted multifunctional nanomicelles for dual-modality imaging and synergistic high-intensity focus ultrasound (Hifu) ablation on pancreatic cancer in nude mice. J Mater Chem B. (2024) 12:5884–97. doi: 10.1039/d4tb00091a. PMID: 38775254

[197] Eranki A Srinivasan P Ries M Kim A Lazarski CA Rossi CT . High-intensity focused ultrasound (Hifu) triggers immune sensitization of refractory murine neuroblastoma to checkpoint inhibitor therapy. Clin Cancer Res. (2020) 26:1152–61. doi: 10.1158/1078-0432.Ccr-19-1604. PMID: 31615935 PMC9009723

[198] Ashar H Singh A Kishore D Neel T More S Liu C . Enabling chemo-immunotherapy with Hifu in canine cancer patients. Ann BioMed Eng. (2024) 52:1859–72. doi: 10.1007/s10439-023-03194-1. PMID: 37162696 PMC12323608

[199] Zhang L Xu C Li M Lu X Sheng Y Chen L . Gambogic acid based coordination polymer reinforces high-intensity focused ultrasound treatment of gynecologic Malignancies. Adv Mater. (2025) 37(26):e2501664. doi: 10.1002/adma.202501664. PMID: 40223396

[200] Su S Wang Y Lo EM Tamukong P Kim HL . High-intensity focused ultrasound ablation to increase tumor-specific lymphocytes in prostate cancer. Transl Oncol. (2025) 53:102293. doi: 10.1016/j.tranon.2025.102293. PMID: 39862483 PMC11803900

